# Cardiomyopathy related desmocollin-2 prodomain variants affect the intracellular cadherin transport and processing

**DOI:** 10.3389/fcvm.2023.1127261

**Published:** 2023-05-19

**Authors:** Greta Marie Pohl, Manuel Göz, Anna Gaertner, Andreas Brodehl, Tolga Cimen, Ardan M. Saguner, Eric Schulze-Bahr, Volker Walhorn, Dario Anselmetti, Hendrik Milting

**Affiliations:** ^1^ Erich & Hanna Klessmann-Institute for Cardiovascular Research and Development & Clinic for Thoracic and Cardiovascular Surgery, Heart- and Diabetes Center NRW, D-32545 Bad Oeynhausen, University Hospital of the Ruhr-University Bochum, Bad Oeynhausen, Germany; ^2^ Experimental Biophysics and Applied Nanoscience, Faculty of Physics, University of Bielefeld, NRW, Bielefeld, Germany; ^3^ Department of Cardiology, University Heart Center Zurich, University Hospital Zurich, Zürich, Switzerland; ^4^ Department of Cardiovascular Medicine, Institute for Genetics of Heart Diseases (IfGH), University Hospital Münster, Münster, Germany

**Keywords:** desmosome, desmocollin-2, DSC2, prodomain, signal peptide, genetic variants, intracellular transport, arrhythmogenic cardiomyopathy

## Abstract

**Background:**

Arrhythmogenic cardiomyopathy can be caused by genetic variants in desmosomal cadherins. Since cardiac desmosomal cadherins are crucial for cell-cell-adhesion, their correct localization at the plasma membrane is essential.

**Methods:**

Nine desmocollin-2 variants at five positions from various public genetic databases (p.D30N, p.V52A/I, p.G77V/D/S, p.V79G, p.I96V/T) and three additional conserved positions (p.C32, p.C57, p.F71) within the prodomain were investigated *in vitro* using confocal microscopy. Model variants (p.C32A/S, p.V52G/L, p.C57A/S, p.F71Y/A/S, p.V79A/I/L, p.I96l/A) were generated to investigate the impact of specific amino acids.

**Results:**

We revealed that all analyzed positions in the prodomain are critical for the intracellular transport. However, the variants p.D30N, p.V52A/I and p.I96V listed in genetic databases do not disturb the intracellular transport revealing that the loss of these canonical sequences may be compensated.

**Conclusion:**

As disease-related homozygous truncating desmocollin-2 variants lacking the transmembrane domain are not localized at the plasma membrane, we predict that some of the investigated prodomain variants may be relevant in the context of arrhythmogenic cardiomyopathy due to disturbed intracellular transport.

## 1. Introduction

Desmosomes are multiprotein complexes that maintain the structure of a tissue that is exposed to high mechanical stress. This intercellular adhesion is mediated by calcium-dependent cadherins. Varying expression patterns and functional differences of cadherins lead to four subclasses: (I) classical (e.g., N-, R-, E-Cadherin), (II) proto-cadherins, (III) desmosomal cadherins (Desmoglein, Desmocollin) and (IV) cadherin-related proteins (e.g., the unique T-Cadherin) (1, 2). Desmosomal cadherins are type-I transmembrane glycoproteins mediating cell-cell-adhesion and can be found e.g., in cardiac desmosomes which contain the isoforms desmoglein-2 (DSG2) and desmocollin-2 (DSC2). These cadherins are synthesized as precursor proteins containing a signal peptide that guides the nascent protein to the endoplasmic reticulum (ER) and a propeptide or a prodomain inhibiting biological cadherin activation during protein maturation. The propeptide or prodomain is cleaved in the late Golgi and finally the mature cadherin is transported to the plasma membrane (3, 4). The prodomain contains numerous amino acids that are highly conserved across species and among different cadherins. The molecular function of these amino acids is not precisely known.

Amino acid alignments of the prodomain reveal high sequence homologies between classical E- (CDH1) (5, 6), N- (CDH2) (7, 8), R- (CDH4) (9), T-cadherins (CDH13) (10) and the desmosomal cadherins like e.g., DSC2 (11, 12). Recently, protein structures of the extracellular cadherin domains (EC) 1 and EC2–5 were published for DSC2 as well as for other desmosomal cadherins (13). However, the molecular structure of the DSC2 prodomain is not precisely known. Of note, the prodomain of N-cadherin has an extracellular cadherin domain-like fold structure lacking any adhesive function (14) which shows striking similarities to the predicted structure of the DSC2 prodomain (15, 16).

Variants in genes encoding desmosomal cadherins are associated with arrhythmogenic cardiomyopathies (ACM, OMIM #125645, #125671) (17), but their pathogenic impact is obviously difficult to interpret in the clinical context. This is due to the limited availability of functional data of these proteins and the lack of large pedigrees for co-segregation analyses to prove the association between ACM and genetic findings in genes encoding desmosomal cadherins. Especially homozygous truncating variants in DSG2 (18) and DSC2 (19–24) were found to be disease-causing. At present it is unknown if some of the reported rare missense variants cause monogenic cardiomyopathies, contribute to the onset of the disease (25, 26) or are even rare variants without any pathogenic impact. Consequently, most variants within the prodomain of DSC2 are assigned as variants with unknown significance (VUS) according to the guidelines of the American College of Medical Genetics and Genomics (ACMG) (27). The majority of database-listed missense variants were functionally uncharacterized.

Therefore, we investigated the *in vitro* effect of nine missense variants, which are listed in genetic databases and affect conserved regions of the DSC2 prodomain. In addition, 14 conserved amino acids that are hypothesized to influence the intracellular localization due to different physicochemical properties of the amino acid residues were generated as model variants. Furthermore, we investigated the physiological role of the wildtype DSC2 signal peptide and the prodomain for the transport of DSC2 in cell culture. We revealed that the prodomain and conserved positions within the prodomain are critical for the subcellular transport of DSC2 to the plasma membrane. In addition, we found evidence that variants at conserved positions within the prodomain may influence the prodomain cleavage. Of note, some variants were transported to the plasma membrane indicating that the position of the amino acid and the physicochemical properties are relevant for the intracellular protein transport.

## 2. Materials and methods

### 2.1. Experimental design and investigated variants

**Figure 1:**
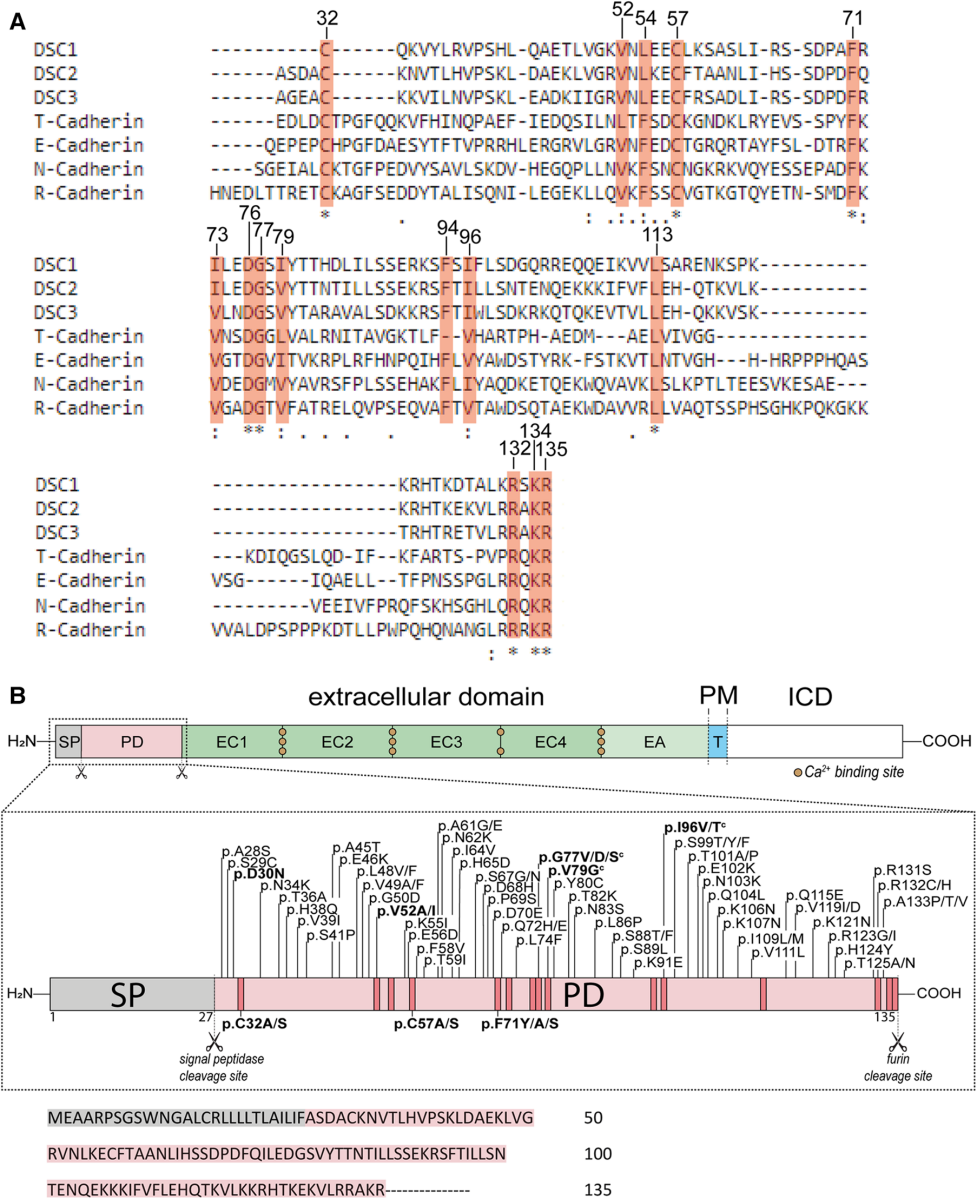
(**A**) comparison of the prodomains (PD) of DSC1-3 and different classical cadherins (N-, R-, E-Cadherin) and cadherin-like proteins (T-Cadherin) from *Homo sapiens* using Clustal Omega (28). Conserved amino acids are marked with orange boxes. Positions on top refer to the open reading frame (ORF) of DSC2. (**B**) **Top**: Schematic overview of the structure of DSC2. The extracellular domain consists of the signal peptide (SP, grey box), the PD (light orange box), four extracellular cadherin domains (EC1-4, green boxes) and an anchor domain (EA, light green box) which is sometimes assigned as a fifth cadherin domain (EC5). A single transmembrane domain (T, blue box) connects the extracellular region with the intracellular domain (ICD, white box). PM, plasma membrane. **Middle**: Schematic overview of the SP and PD of DSC2. Missense variants that are associated with ACM are shown on top and were received from the ARVC database (29), the Human Gene Mutation Database (HGMD) (30), from the National Library of Medicine ClinVar (31) and the genome aggregation database (gnomAD) (32). Variants below are investigated model mutants. All variants are classified as variants with unknown significance (VUS) according to the ACMG guidelines (27). Variants shown in bold letters were investigated. Scissors represent cleavage sites. Orange boxes mark highly conserved amino acids. **^c^** indicates variants that share conflicting interpretation of pathogenicity in various genetic databases. **Bottom**: Primary amino acid sequence of the SP and PD of human DSC2.

Amino acid alignments of the prodomain of DSC2 with other cadherins from two different cadherin families (classical and cadherin-like proteins) and different mammalian species revealed a high degree of homologies (Figure 1A and Supplementary Figure S1). The sequences for the alignments were obtained from the database UniProt (33).

Some of the DSC2 prodomain variants listed in the genetic disease databases affect conserved amino acids and may have impact on its function. We selected missense variants at phylogenetically conserved positions from ClinVar (31), the Human Gene Mutation Database (HGMD) (30) and the ARVC database (29) (Table 1 and Figure 1B). Furthermore, three variants affecting conserved positions (p.V52I, p.G77V, p.I96V) were obtained from the population Genome Aggregation Database (gnomAD) (32) and two of those (p.G77V, p.I96V) were also listed as VUS in ClinVar (Table 1).

**Table 1:** Overview of the investigated variants in this study. For each variant the nucleotide (nt) change, the reference SNP (rs), the minor allele frequency (MAF) as well as the classification and the database/s is/are given when present.

Variant	nt-exchange	Reference SNP	MAF°	ACMG-Classification
p.D30N	c.88G > A	rs1987673824	–	VUS^1^
p.C32S	c.94T > G, c.95T > C	model variant	–	–
p.C32A	c.95T > C	model variant	–	–
p.V52A	c.155T > C	rs1987629779	–	VUS^1^
p.V52I	c.154G > A	rs757588306	0.0004	VUS^2^
p.V52G	c.155T > G	model variant	–	–
p.V52l	c.154G > C	model variant	–	–
p.C57S	c.170G > C	model variant	–	–
p.C57A	c.169T > G, c.170G > C	model variant	–	–
p.F71Y	c.212T > A	model variant	–	–
p.F71A	c.211T > G, c.212T > C	model variant	–	–
p.F71S	c.212T > C	model variant	–	–
p.G77V	c.230G > T	rs761544006	0.0004	VUS^1^^,^^2^
p.G77D	c.230G > A	rs761544006	–	VUS^1^
p.G77S^c^	c.229G > A	–	–	VUS^3^, pathogenic^4^^,^^**^
p.V79A	c.236T > C	model variant	–	–
p.V79I	c.235G > A	model variant	–	–
p.V79G^c^ (34)	c.236T > G	rs886038828	–	VUS^1^, pathogenic^4^
p.V79l	c.235G > C	model variant	–	–
p.I96V	c.286A > G	rs772447450	0.002	VUS^1^^,^^2^
p.I96T^c^	c.287T > C	rs1320040048	–	pathogenic^3^^,^^4^^,^^**^
p.I96l	c.286A > C	model variant	–	–
p.I96A	c.286A > G, c.287T > C	model variant	–	–

^1^ ClinVar (31)

^2^ gnomAD (32)

^3^ ARVC database (29)

^4^ HGMD (30)

^c^ conflicting interpretation (08/2022), **−**, no data available, °MAF; derived from gnomAD (10/2022)

^**^ Classification is based on *in silico* data (SIFT (35), PolyPhen (36)) only (03/2023).

The *in vitro* effects of the selected DSC2 variants affecting phylogenetically conserved amino acids at p.V52, p.G77, p.I96 (among others from gnomAD) and in addition p.V79 (HGMD and ClinVar) (34) were investigated (Table 1). We excluded the variants p.R132C (ClinVar, HGMD, ARVC database, gnomAD) and p.R132H (ClinVar, HGMD gnomAD) since they affect the DSC2 prodomain cleavage site (37). Variants in this motif inhibit the cleavage of the propeptide in DSG2 (38, 39).

In addition, the variant DSC2-p.D30N located in a less conserved position within the prodomain is listed in ClinVar as VUS (Table 1). We investigated this variant since it may affect the predicted signal peptidase cleavage site (SignalP v. 6.0; Supplementary Figure. S2) (40).

Furthermore, we included in our study the conserved positions p.C32, p.C57 and p.F71 within the prodomain of DSC2 and exchanged the respective amino acids to investigate the impact on their membrane localization. Using cell transfection experiments in combination with confocal microscopy we investigated the *in vitro* effects of the various missense variants. The wildtype DSC2 and the variants p.D30N and p.V79G were also tested in a secretion assay.

**Figure 2:**
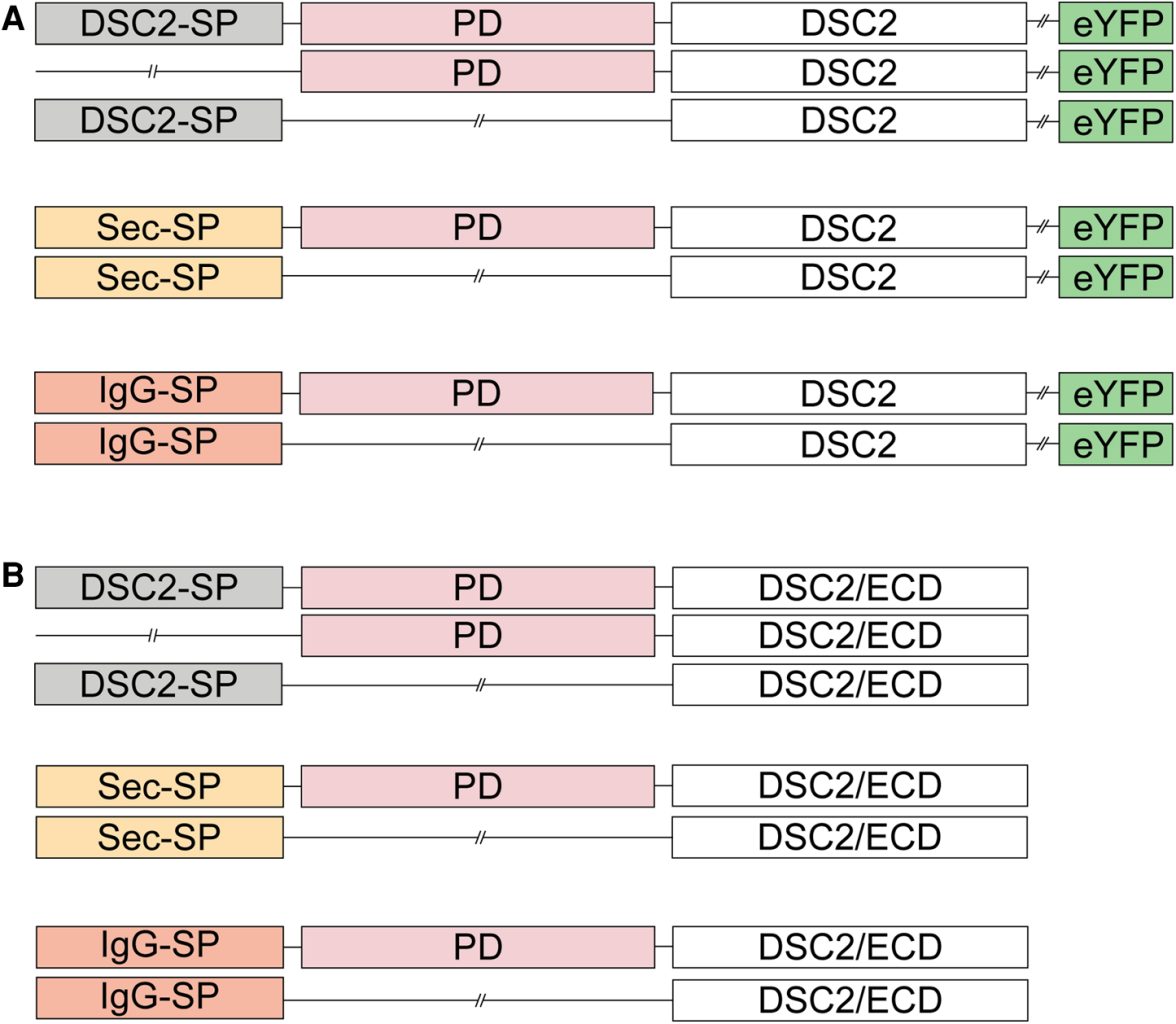
Schematic overview of the DSC2 constructs used for transient transfection of (**A**) HT-1080 cells and hIPSC-derived cardiomyocytes for confocal microscopy and (**B**) HEK293 c18 cells for the secretion assay. SP, signal peptide; PD, prodomain; eYFP, enhanced yellow fluorescence protein; DSC2/ECD, extracellular cadherin domain of desmocollin-2.

Furthermore, the role of the DSC2 signal peptide and the prodomain was investigated in different cell lines. We generated DSC2 constructs (Figure 2A) with and without the wildtype signal peptide lacking the prodomain. We also generated DSC2 constructs with and without the prodomain combined with the signal peptides of the immunoglobulin heavy chain (IgG; MEFGLSWLFLVAFLKGVQC) (13) or the ideal human secretory signal peptide (Sec; MWWRLWWLLLLLLLLWPMVWA), which was predicted by the Hidden Markov Model (41, 42).

### 2.2. Plasmid generation and site-directed mutagenesis

The peYFP-N1-wildtype plasmid of DSC2 was previously described (43). For the secretion assay the cDNA encoding the extracellular domains of DSC2 (aa1-aa694) was cloned into pCEP4-vector (Addgene plasmid #53050; kindly provided by Dr. Michael Sanguinetti) (44). The N-terminal Kozak sequence (5′-GCCGCCACC-3′) was added by PCR using appropriate primers. Variants were introduced into both plasmids using the QuikChange Lightning Site-Directed Mutagenesis Kit (Supplementary Table S1; Agilent Technologies, Santa Clara, CA, USA). The DSC2 encoding parts of all plasmids were verified via Sanger sequencing (Macrogen, Amsterdam, Netherlands) (45). Sequences encoding the alternative signal peptides were cloned using CloneJET PCR Cloning Kit (Thermo Fisher Scientific, Waltham, MA, USA) according to the manufacturer’s protocol.

### 2.3. Cell culture

Cells (HT-1080, DSMZ-German Collection of Microorganisms and Cell Cultures, Brunswick, Germany, #ACC315; HEK293 c18, ATCC, Manassas, VA, USA, CRL-10852), which do not express endogenous DSC2 (46, 47) were cultured in Dulbecco's Modified Eagle Medium (DMEM, 4.5 g/L glucose, Thermo Fisher Scientific) supplemented with 10% fetal bovine serum (FBS) and 1% penicillin and streptomycin (Sigma-Aldrich, St. Louis, MO, USA) under standard conditions (37°C, 5% CO_2_). The medium for HEK293 c18 was additionally supplemented with 0.25 mg/ml G418 (Thermo Fisher Scientific).

HiPSCs (NP0040-8, UKKi011-A, kindly provided by Dr. Tomo Saric, University of Cologne, Germany) were cultured in Essential E8 Medium (Thermo Fisher Scientific) and differentiated into cardiomyocytes as previously described (25, 48).

For confocal microscopy the human fibrosarcoma cell line HT-1080 was cultured in 8-well µ-slide cover slides (Ibidi, Gräfelfing, Germany). HiPSC-derived cardiomyocytes were cultured in Matrigel-coated 8-well µ-slide cover slides (Ibidi).

For the secretion assay HEK293 c18 cells containing EBNA1 were cultured in 6-well plates to obtain high and efficient expression of recombinant cadherins (49).

### 2.4. Transient transfection

For confocal microscopy HT-1080 cells were transfected at 80% confluency with 500 ng DNA using Lipofectamin LTX (Thermo Fisher Scientific) according to the manufacturer's protocol (ratio 1:2 Plasmid:Lipofectamin LTX). HEK293 c18 cells were transfected at 100% confluency corresponding to 1.2*10^6^ cells with 2.5 µg DNA by using Opti-MEM (Thermo Fisher Scientific) and linear polyethylenimine (PEI, Sigma-Aldrich) (ratio 1:3 Plasmid:PEI) (50) for the secretion assay.

HiPSC-derived cardiomyocytes were transfected using Lipofectamin 3000 (ratio 1:2 Plasmid:Lipofectamin 3,000) (Thermo Fisher Scientific) according to the manufacturer's protocol. Four hours prior transfection the medium was changed to RPMI 1,640 (Thermo Fisher Scientific) supplemented with B-27 (#17504044, Thermo Fisher Scientific) and 10% FBS.

As negative controls only the transfection reagents and Opti-MEM were added to the cells to estimate the effects of cell toxicity due to transfection and are further indicated as non-transfected (NT).

### 2.5. Secretion assay

Ectodomain constructs of DSC2 with different signal peptide or prodomain variants (Figure 2B) were used to obtain secretion of the truncated proteins into the cell culture supernatant. The secretion of the protein construct was analyzed by Western blotting.

24 h post transfection the medium was changed to serum-free FreeStyle293 Expression Medium (Thermo Fisher Scientific) and 48 h post medium change the supernatants were collected and centrifuged at 18,500×g for 10 min at 4°C. In parallel, cell lysates were generated. Cells were washed twice gently with 1× Dulbecco's phosphate buffered saline (PBS, Thermo Fisher Scientific). 100 µl RIPA Lysis buffer (150 mM NaCl, 25 mM Tris-HCl, 1% Nonidet-P40, 0.5% Sodium deoxycholate, 0.1% SDS) were added and the cells were harvested using a cell scraper (Sarstedt, Nümbrecht, Germany). After three freeze-thaw cycles in liquid nitrogen the cells were sonicated for 1 min. Finally, the lysates were centrifuged at 18,500×g for 10 min at 4°C to remove cell debris. The supernatants and lysates were analyzed by sodium dodecyl sulfate polyacrylamide gel electrophoresis (SDS-PAGE) and Western blotting.

### 2.6. Western blot analyzes

Equal volumes of the cell culture supernatants and lysates (10 µl) were resuspended in 6× Laemmli buffer (51), denatured at 95°C for 5 min and separated in Mini-Protean 4%–20% TGX gels (15-well, Bio-Rad, Hercules, CA, USA). Blotting was done by using the Trans-Blot Turbo Transfer System (Bio-Rad) at 25 V for 3 min. Afterwards, nitrocellulose membranes were blocked with 5% (w/v) skimmed milk in Tris-buffered saline/1% (v/v) Tween-20 (TTBS) at 60 rpm on a horizontal shaker for 1 h at room temperature (RT). After washing three times for 10 min with TTBS, the membranes were incubated with primary antibody against DSC2 (1:1,000 in TTBS, 4°C overnight, #72792, Abcam, Cambridge, UK). For loading control anti-glyceraldehyde-3-phosphate dehydrogenase antibody (GAPDH; 1:1,000 in TTBS, 4°C overnight, #9485, Abcam) was used. After washing three times with TTBS the membranes were incubated with the secondary antibody [Horseradish Peroxidase (HRP) coupled anti-mouse-IgG, 1:5,000 in TTBS, RT, 1 h, #554002, BD Biosciences, Franklin Lakes, USA]. For the loading control the anti-rabbit-IgG (HRP-conjugated, 1:5,000 in TTBS, RT, 1 h, NA934V, Sigma-Aldrich) was used. The membranes were washed three times with TTBS and were developed with the WesternBright Quantum Kit (Advansta, San Jose, CA, USA) according to the manufacturer's protocol. Documentation was done in the FluorChem2 Imaging System (Intas, Göttingen, Germany).

### 2.7. Immunocytochemistry

Cells were stained 24 h post transfection. Therefore, HT-1080 cells and hiPSC-derived cardiomyocytes were washed twice with PBS. HT-1080 cells were fixed with 4% paraformaldehyde (PFA) for 10 min at RT prior staining of the plasma membrane using wheat germ agglutinin conjugated to Alexa Fluor 633 (WGA; 1:200 in PBS, 3 min, RT, Thermo Fisher Scientific). The plasma membrane of hiPSC-derived cardiomyocytes was stained using WGA conjugated to Alexa Fluor 647 (1:200 in PBS, 3 min, RT, Thermo Fisher Scientific) prior fixation (4% PFA, 10 min at RT).

The endoplasmic reticulum (ER) and Golgi apparatus were tracked in HT-1080 cells. In hiPSC-derived cardiomyocytes the sarcomeric α-actinin (ACTN2) was stained. Therefore, cells were washed twice and permeabilized for 10 min with 0.1% (v/v) Triton X-100/PBS (Sigma-Aldrich) at RT. After gentle washing twice with PBS, the cells were blocked with 5% (w/v) BSA/PBS for 1 h at RT.

Antibodies against the ER protein Calnexin (CANX; 1:100 in PBS, overnight, 4°C, PA5-34754, Thermo Fisher Scientific) and N-acetylgalactosaminyltransferase 2 (GALNT2; 1:500 in PBS, overnight, 4°C, #262868, Abcam, Cambridge, UK) as a marker for the Golgi apparatus were used. As secondary antibody Cy3-conjugated anti-rabbit IgG (1:100 in PBS, 1 h, RT, C2306, Sigma Aldrich) was used.

HiPSC-derived cardiomyocytes were incubated with primary antibody against ACTN2 [1:100 in 1% (w/v) BSA/PBS, 4°C, overnight, #A7732, Sigma-Aldrich]. After washing twice with PBS, the cells were incubated with the secondary antibody against mouse IgG conjugated with Cy3 (1:100 in PBS, #115-165-068, Jackson ImmunoResearch, Ely, UK).

Nuclei were co-stained for 5 min at RT using 4′,6-diamidino-2-phenylindole (DAPI, 1 µg/ml). The cells were stored in PBS at 4°C until microscopy was performed.

### 2.8. Confocal microscopy

For confocal microscopy the TCS SP8 system in combination with the Leica Application Suite X (LasX) software were used (Leica Microsystems, Wetzlar, Germany). Cy3 was excited at 552 nm, eYFP at 488 nm and DAPI at 405 nm. Alexa Fluor 633 and 647 were excited at 638 nm.

Fluorescence emission was detected in the range of 493–548 nm for eYFP, 551–596 nm for Cy3, 410–460 nm for DAPI and 643–784 nm for Alexa Fluor 633 and 647. The fluorescence dyes were sequentially excited.

### 2.9. Modelling of different variants in the prodomain of DSC2

Signal- and prodomain-sequences were used for modelling using ColabFold (52). Information on intramolecular bonds of the wildtype were taken from the AlphaFold Database (alphafold.ebi.ac.uk/entry/Q02487) (15, 16). From five models the model with the highest predicted Local Distance Difference Test per position (pLDDT) (53) and lowest Predicted Aligned Error (PAE) was used for further analysis using PyMOL, version 2.5.2 (Schrödinger). For the construction of intramolecular contacts in PyMOL “polar contacts to other atoms in object” was selected for the respective amino acid.

### 2.10. Statistical analysis

Western blotting was performed in triplicates (*n* = 3). Each fluorescence analysis was performed in at least quadruplicates (*n* = 4).

Per transfection experiment at least 100 cells were manually analyzed blinded for localization or absence of the eYFP-conjugated DSC2 at the plasma membrane.

For statistical evaluation GraphPad Prism Version 9.0 (GraphPad Software, San Diego CA, USA) the nonparametric Kruskal-Wallis test followed by Dunn's multiple comparison was used. A *p*-value <0.05 was considered significant. All data are presented as mean ± standard deviation (SD).

### 2.11. Quantification of colocalization

To quantify the colocalization LasX (Leica Microsystems) software was used to determine the Pearson's correlation coefficient (PCC) (54). At least three cells were used for quantification. Per cell a region of interest of 100 µm^2^ was used for evaluation.

## 3. Results

### 3.1. The DSC2 prodomain is essential for intracellular transport to the plasma membrane

**Figure 3:**
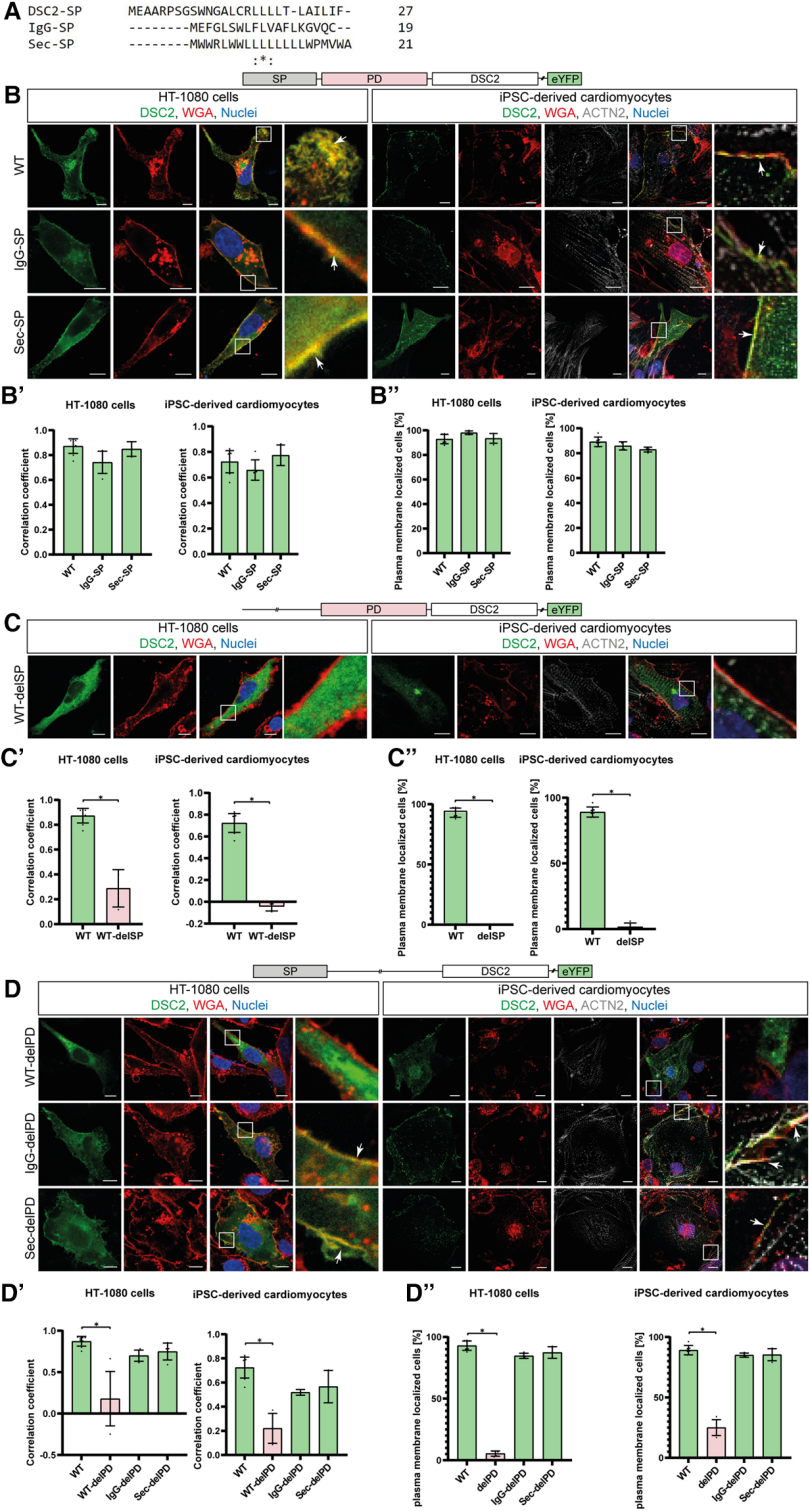
Influence of alternative signal peptides (SP) on the localization of human DSC2. (**A**) Amino acid alignment of the three different SPs (DSC2/IgG/Sec-SP) using Clustal Omega (28). (**B**) Representative images of the construct localization with different SPs, (**C**) DSC2 lacking the SP and (**D**) constructs lacking the DSC2 prodomain (PD). HT-1080 cells and hiPSC-derived cardiomyocytes were transiently transfected with different eYFP-conjugated DSC2 constructs (green). Wheat germ agglutinin (WGA) conjugated to Alexa Fluor 633 (for HT-1080) and Alexa Fluor 647 (for hiPSC-derived cardiomyocytes) was used as a plasma membrane marker and is shown in red. Anti α-Actinin (ACTN2) antibody was used as a cardiomyocyte marker (grey). Nuclei were co-stained with 4′,6-diamidino-2-phenylindole (DAPI, blue). Arrows indicate the colocalization of DSC2 and WGA. Scale bars represent 10 µm. For each construct Pearson correlation coefficient (B’, C’, D’) was determined with a region of interest of 100 µm^2^. At least 100 cells were manually counted blinded for plasma membrane localization by an investigator (B’’, C’’, D’’). Nonparametric Kruskal-Wallis test followed by Dunn's multiple comparison was performed using GraphPad Prism Version 9.0. *P*-values <0.05 were considered significant (*). Mean values ± standard deviation are shown. Of note, wildtype and alternative SP constructs are localized at the plasma membrane. Without the SP or prodomain DSC2 is not properly localized. Interestingly, with IgG- or Sec-SP but lacking the PD the protein is localized at the plasma membrane as the wildtype.

We analyzed N-terminal deletion mutants to prove the functional role of DSC2 signal peptide and prodomain. In addition, we combined different signal peptides including the wildtype signal peptide with or without the prodomain of DSC2 (Figure 2A). DSC2-eYFP constructs lacking the signal peptide (WT-delSP) or the prodomain (WT-delPD) were expressed but were not transported to the plasma membrane. Of note, all constructs with alternative signal peptides (IgG/Sec-SP, IgG/Sec-delPD) were expressed and transported to the plasma membrane irrespective of the presence of the prodomain (Figures 3, 5A,B).

### 3.2. Missense variants related to arrhythmogenic cardiomyopathies within the DSC2 prodomain can affect the intracellular transport of the cadherin to the plasma membrane

**Figure 4:**
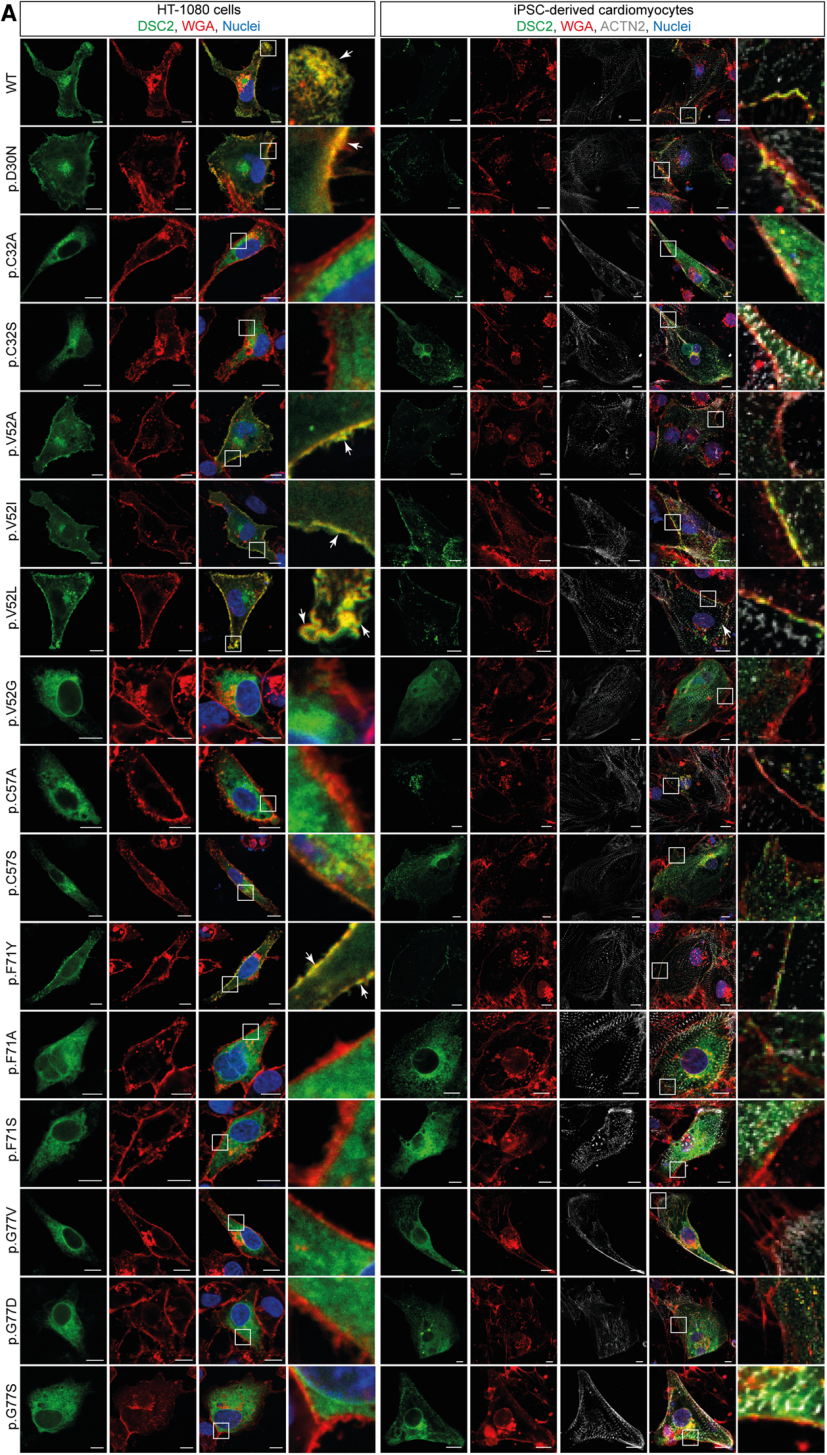

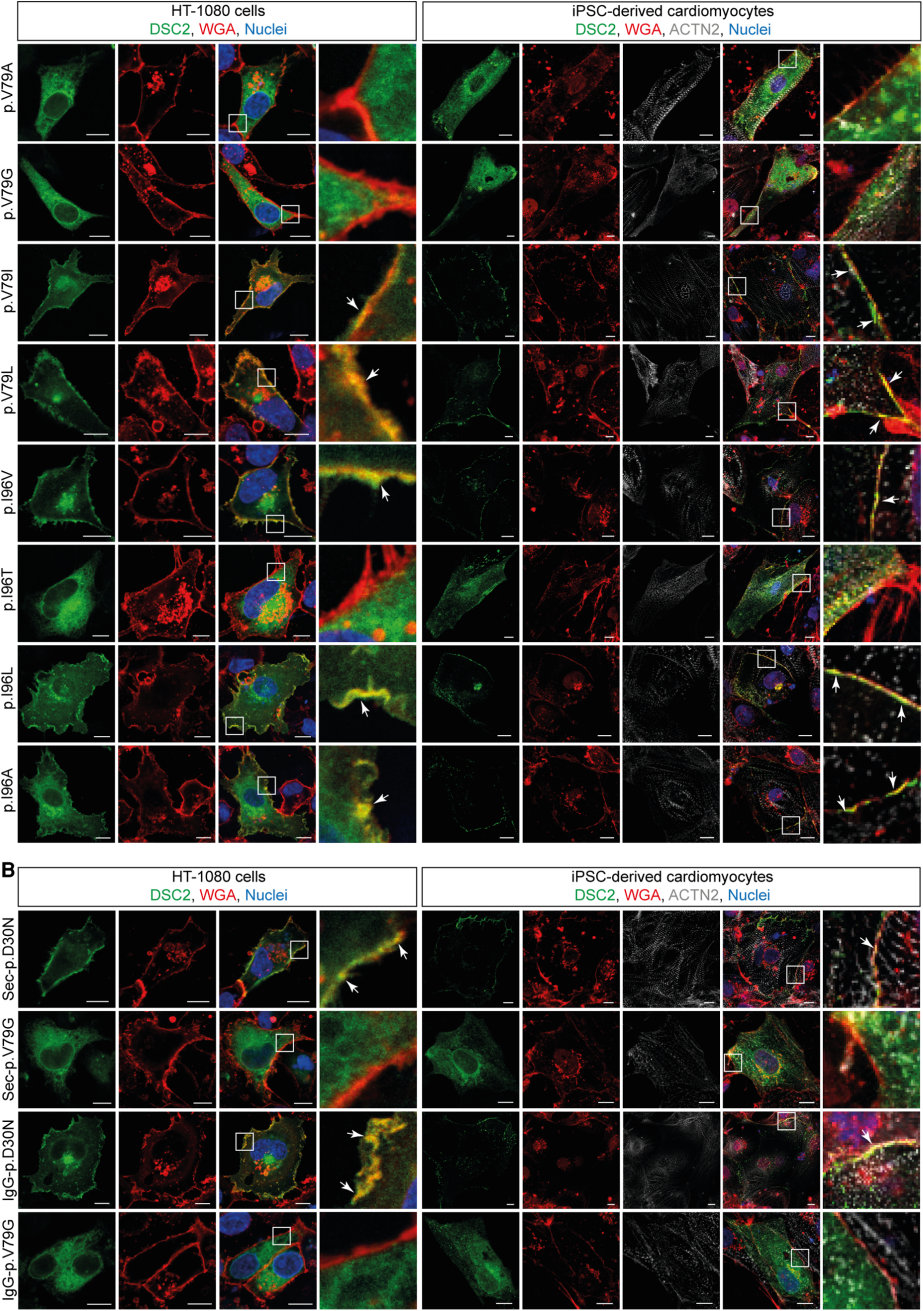

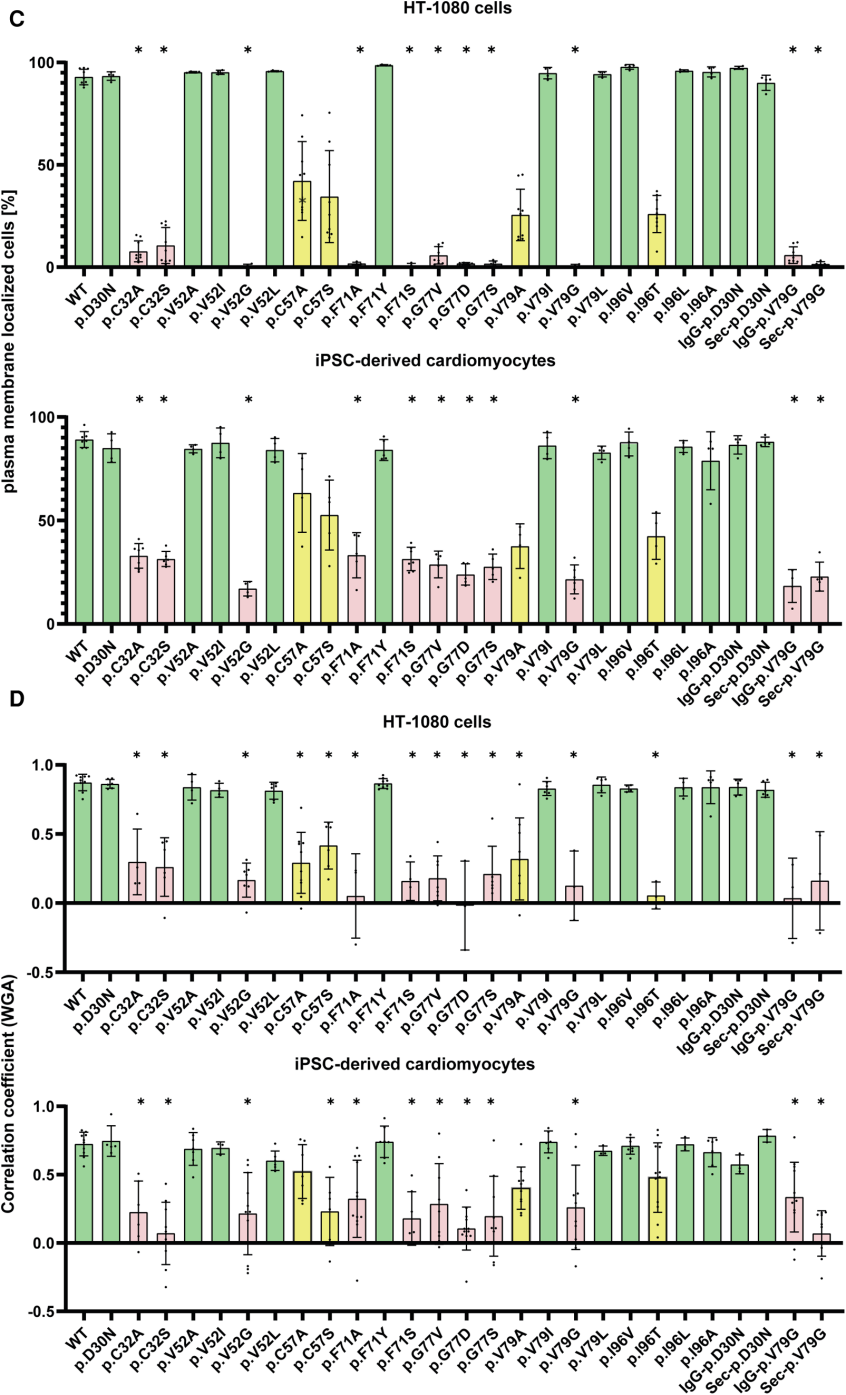

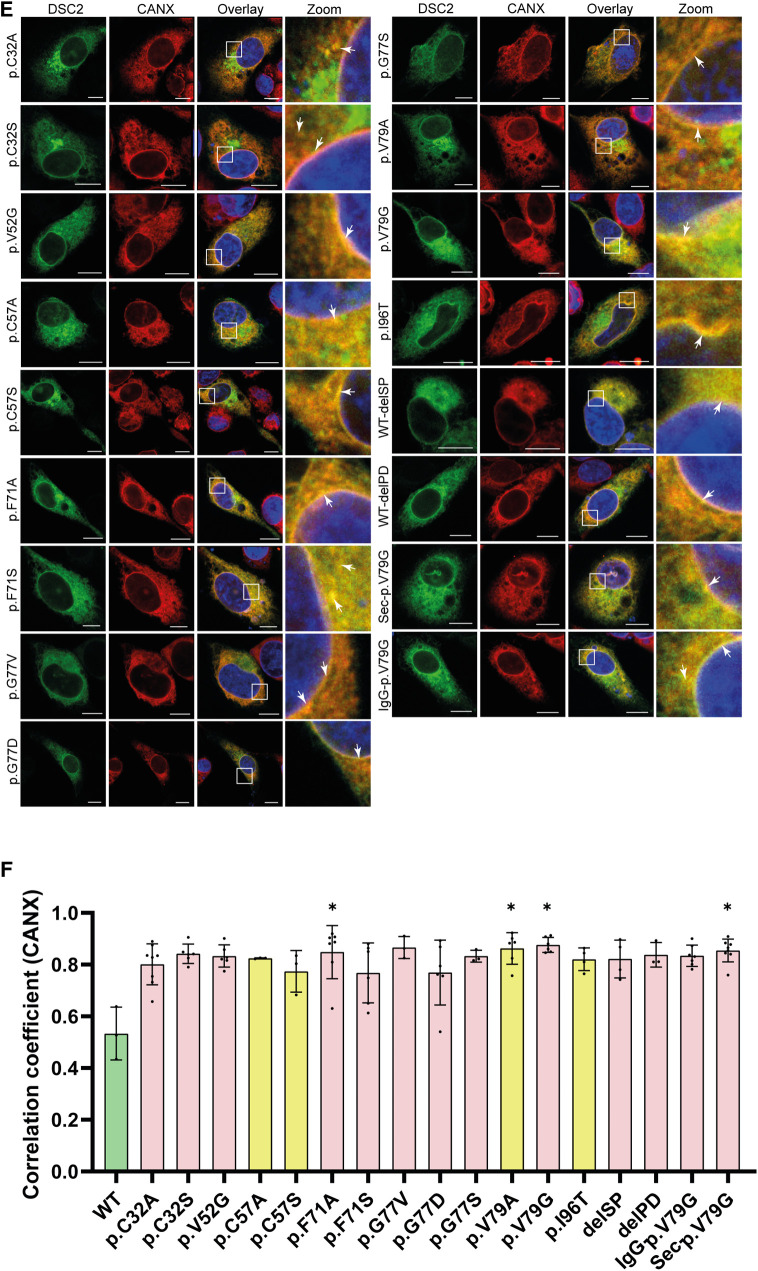

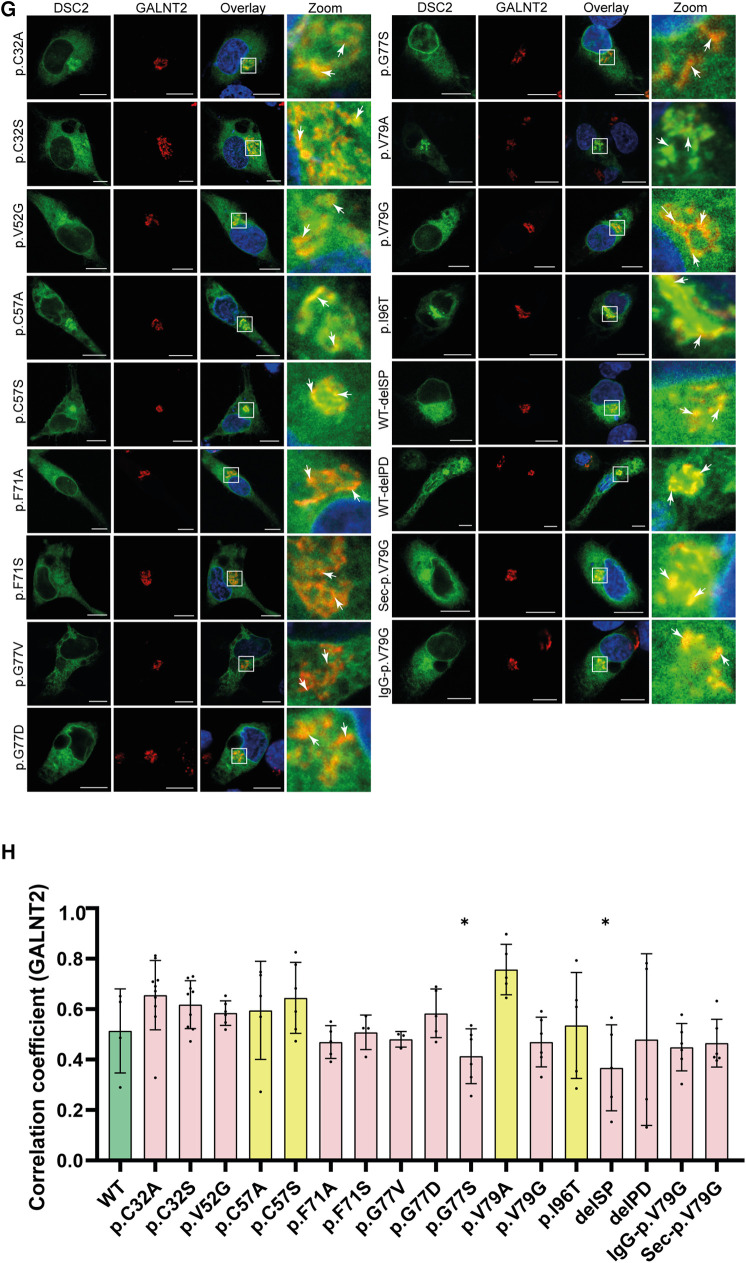
Representative images of HT-1080 cells and hiPSC-derived cardiomyocytes transiently transfected with different eYFP-conjugated DSC2 constructs. (**A**) Prodomain constructs in conserved positions of DSC2 (green). (**B**) Two representative prodomain variants (p.D30N, p.V79G) with alternative signal peptides IgG- or Sec-SP. The plasma membrane was co-stained with wheat germ agglutinin conjugated with Alexa Fluor 633 (for HT-1080) and Alexa Fluor 647 (for hiPSC-derived cardiomyocytes) (red). Anti α-Actinin (ACTN2) antibody was used as a cardiomyocyte marker (grey). (**C**) Statistics of DSC2 prodomain constructs localized at the plasma membrane *in vitro* and (**D**) Pearson correlation coefficient for plasma membrane localization of DSC2 prodomain constructs. Organelles were tracked using (**E**) anti Calnexin (CANX) antibody for co-staining of the ER and (**G**) anti N-acetylgalactosaminyltransferase 2 antibody (GALNT2) for the Golgi apparatus in HT-1080 cells. Nuclei were stained with 4′,6-diamidino-2-phenylindole (DAPI, blue). Scale bars represent 10 µm. Colocalization of the DSC2 prodomain constructs with the endoplasmic reticulum (ER) and the Golgi apparatus was evaluated by determining Pearson correlation coefficients with a region of interest of 100 µm^2^ (**F,H**). Green bar graphs represent variants that show plasma membrane localization, red bar graphs are variants that do not reach the membrane and yellow bar graphs represent variants that show a tendency to be poorly transported. For statistical analysis nonparametric Kruskal-Wallis test followed by Dunn's multiple comparison was used. *P*-values <0.05 were considered significant (*). Mean values ± standard deviation are shown. DSC2 constructs that are not transported to the plasma membrane remain within the ER or Golgi.

**Figure 5:**
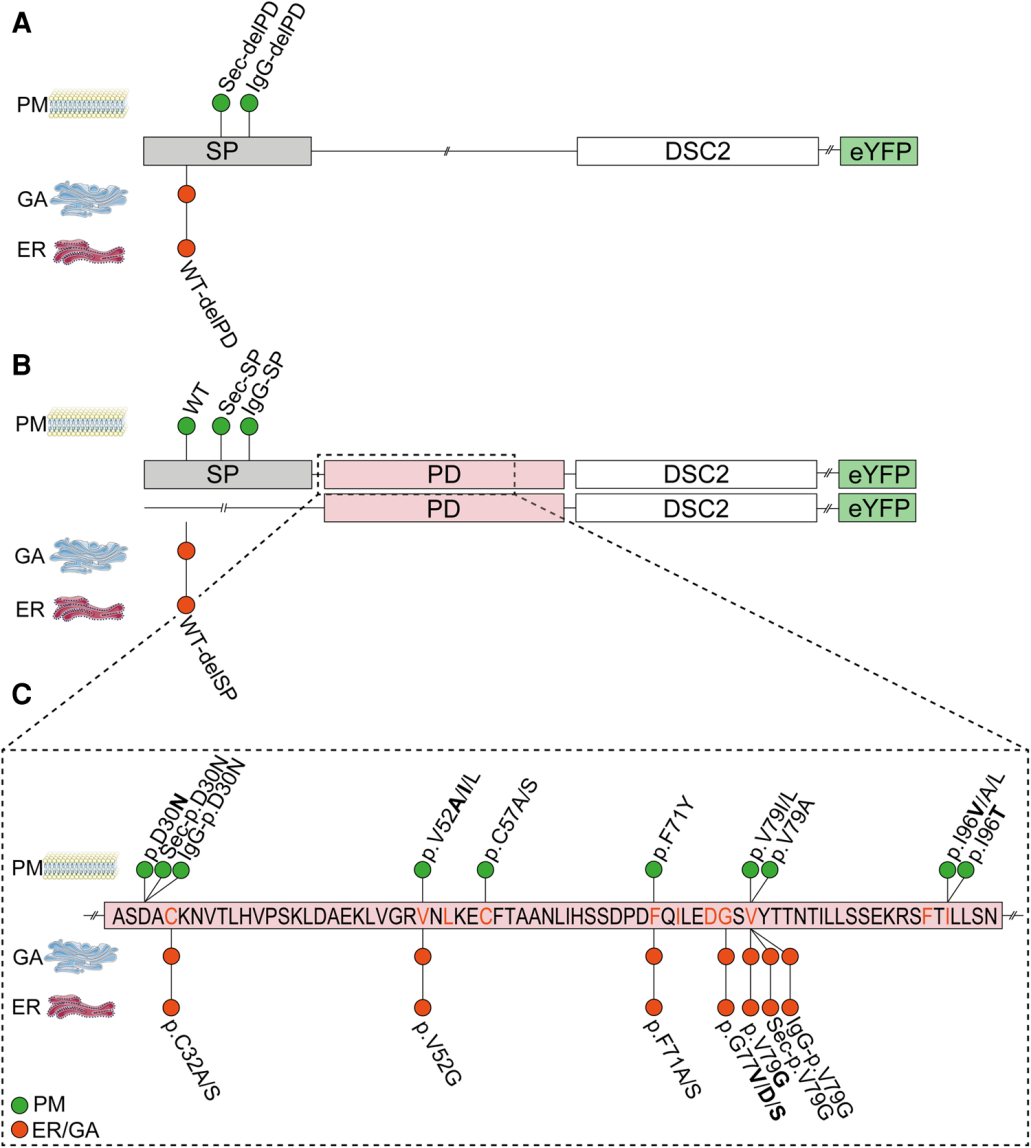
Schematic overview of the localization of different DSC2 constructs *in vitro* in HT-1080 cells and hiPSC-derived cardiomyocytes. (**A**) Different signal peptide (SP) constructs lacking the prodomain (-delPD) show localization with IgG- or Sec-SP but not with wildtype SP. (**B**) Different SP constructs with DSC2 prodomain (PD). Proper localization was observed when wildtype SP and IgG- or Sec-SP were conjugated to the PD. When lacking the SP (WT-delSP) DSC2 was not integrated in the plasma membrane. (**C**) Partial primary sequence of the prodomain of DSC2 (aa27-aa100). Conserved amino acids are given in orange. ACM-related variants are bold. ER, Endoplasmic reticulum; GA, Golgi apparatus; PM, Plasma membrane; WT, wildtype DSC2. Pictures of PM, ER and GA were taken from Servier Medical Art. Servier Medical Art by Servier is licensed under a Creative Commons Attribution 3.0 Unported License (https://creativecommons.org/licenses/by/3.0/).

In total, nine different DSC2 variants derived from genetic databases and located at five phylogenetically conserved prodomain positions were analyzed for their plasma membrane localization (p.D30N, p.V52A/I, p.G77V/D/S, p.V79G, p.I96V/T; Table 1). The variants p.D30N, p.V52A/I are transported to the plasma membrane comparable to wildtype DSC2. All analyzed variants at position 77 (p.G77V/D/S) and p.V79G were expressed but not transported to the plasma membrane remaining within the ER or the Golgi apparatus (Figures 4A,C–H, 5C). Of note, the variants p.I96V/T showed plasma membrane localization but compared to the wildtype the variant p.I96T showed a trend to be poorly transported (Figures 4A,C–H, 5C). Thus, 4 of 9 listed variants were not transported to the plasma membrane.

### 3.3. Phylogenetically conserved amino acids within the prodomain influence the transport of DSC2 to the plasma membrane

We further investigated amino acid exchanges at the conserved positions p.C32, p.C57, p.F71 and variants listed in databases at p.V52, p.V79 and p.I96 for their role on the DSC2 plasma membrane transport. In total, 14 model variants not listed in the genetic databases (p.C32A/S, p.V52G/L, p.C57A/S, p.F71Y/A/S, p.V79A/I/L, p.I96l/A) were generated to investigate the effects of different amino acid side chains (Table 1 and Figures 4A,C–H, 5C).

The variants p.C32S/A were not found at the plasma membrane revealing that the cysteine 32 is critical for the DSC2 transport. In contrast, both variants p.I96l/A were transported like the wildtype. Heterogenous results were observed with exchanges at positions 52, 71 and 79. The exchange of p.V52l was tolerated whereas p.V52G was not found at the plasma membrane. Thus, all branched chain amino acids or alanine at this position (see 4.2) were tolerated with respect to the DSC2 plasma membrane localization. Replacement of p.F71 against tyrosine (p.F71Y) led to plasma membrane localization whereas p.F71A/S disturbed the proper transport of DSC2. In contrast to the disease related missense variant p.V79G, all DSC2 mutants with branched chain amino acids at this position (p.V79I/L) were localized at the plasma membrane. Of note, the exchanges of p.C57S/A and p.V79A did not show a significant difference for their plasma membrane localization compared to the wildtype but a tendency to have a slight impact on the transport could be observed in our *in vitro* data. due to a large spread of the data.

### 3.4. Variants located near the signal peptidase cleavage site may affect the transport of DSC2

The variant p.D30N did not affect a conserved position but may influence the signal peptidase cleavage site, which is predicted *in silico* between positions p.A31-p.C32 (Supplementary Figure S2) (40). A proper localization at the plasma membrane was found for p.D30N indicating no obvious effect on the intracellular DSC2 transport (Figure 4A). In contrast, it is expected that variants at position p.C32 are likely to affect the predicted signal peptidase cleavage site. Consequently, we observed that the variants p.C32A and p.C32S led to a disturbed intracellular protein transport (Figures 4A,C–H, 5C).

### 3.5. The prodomain variant p.V79G affects intracellular DSC2 transport irrespective of the signal peptide

A pathogenic impact of the variant p.V79G was reported in the literature (34), databases (HGMD and ClinVar) and previous experiments (Figure 4). Therefore, we focused on this variant in further experiments. In section 4.1 we showed that constructs lacking the prodomain but coupled with N-terminal alternative signal peptides were found comparable to the wildtype DSC2 at the plasma membrane. Interestingly, when the prodomain containing the p.V79G variant was fused to the N-terminal IgG- or the Sec-signal peptide the plasma membrane localization was disturbed retaining the cadherins within the ER and Golgi apparatus. In contrast, the variant p.D30N did not influence the plasma membrane localization (Figures 4B–H, 5C).

### 3.6. The prodomain missense variant p.V79G is probably not correctly processed

Ectodomain constructs of DSC2 lacking the transmembrane domain were further evaluated using a secretion assay (Figure 2B). Of note, the construct with the native signal peptide lacking the prodomain was not transported whereas the replacement of the wildtype signal peptide by the IgG- or the Sec-signal peptide restored secretion.

**Figure 6:**
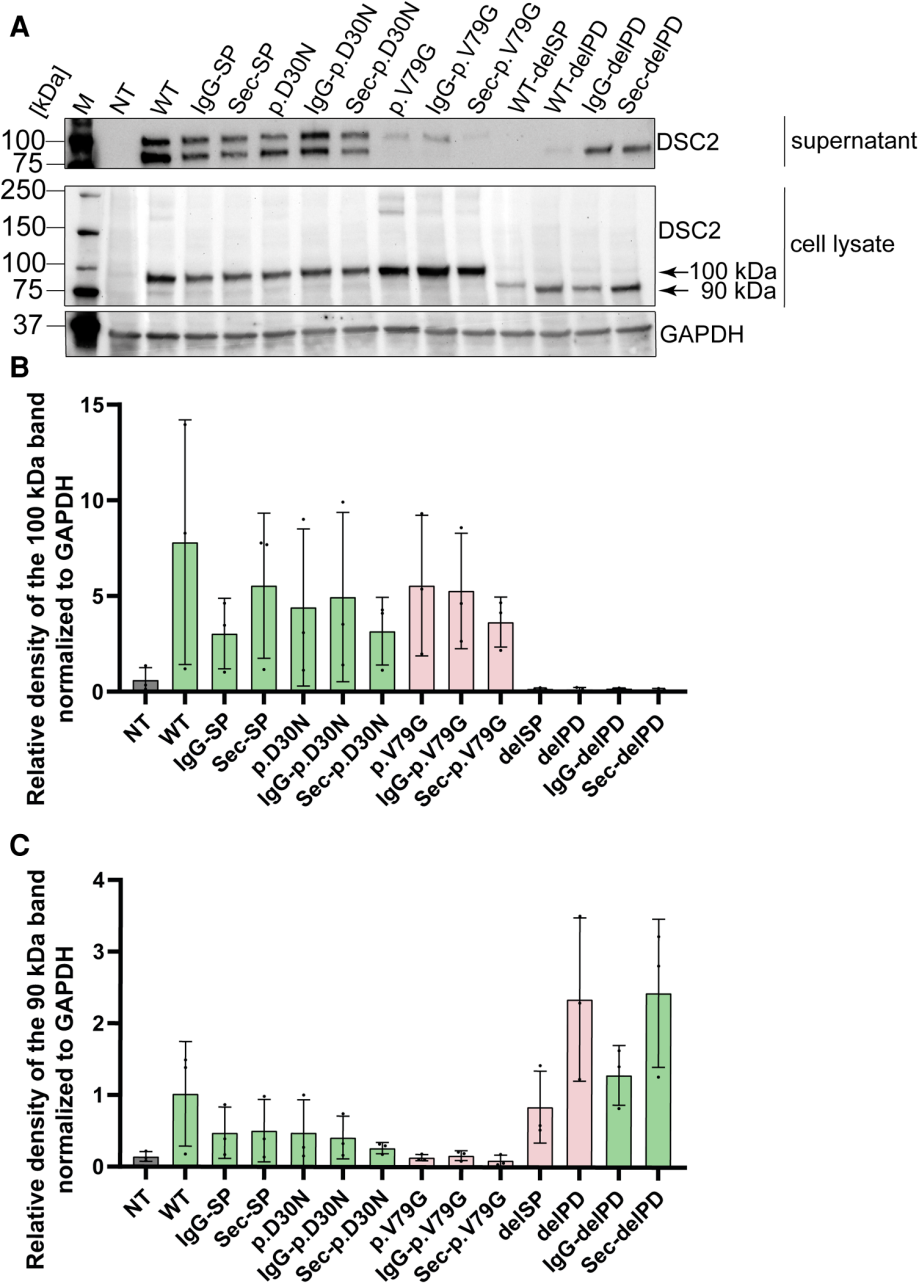
Western blot analysis of secretion assay. (**A**) Cell culture supernatant on top and HEK293 c18 lysate (bottom). Primary antibody was used against DSC2. As loading control for the cell lysate GAPDH was used. Bar graphs showing relative density of DSC2 constructs normalized to GAPDH (**B**) at 100 kDa and (**C**) at 90 kDa. Green bar graphs represent variants which were efficiently expressed and red bar graphs show variants which were expressed but not efficiently secreted. delPD, deleted prodomain of DSC2; delSP, deleted signal peptide of DSC2; M, Marker, NT, non-transfected (negative control); WT, wildtype DSC2.

We observed a translation of the tested prodomain variants, which did not differ significantly compared to the wildtype DSC2. However, the secretion of p.V79G was inefficient suggesting a perturbation of the intracellular transport process (Figure 6). The variant p.V79G showed an apparent molecular mass of 100 kDa in the secretion assay indicating incomplete cleavage of the prodomain. Although it is not clear which enzyme is responsible for the cleavage of the DSC2 prodomain online prediction tools predict furin as a putative protease (55, 56).

Of note, all constructs containing the prodomain or the variant p.D30N but not p.V79G were found at two different molecular masses of approximately 100 and 90 kDa, respectively. In contrast, for constructs lacking the prodomain a single band of 90 kDa was observed (Figure 6). Furthermore, especially for the wildtype DSC2 signal peptide with p.V79G additional bands at 200 and 180 kDa were observed representing presumably intracellular DSC2 dimers (Figure 6).

### 3.7. Molecular modelling of wildtype and mutant prodomains

To investigate possible effects of the variants on the spatial structure, wildtype and mutant DSC2 prodomains were modelled using ColabFold (52). Structural modelling revealed no significant differences in the DSC2 prodomain as compared to the wildtype conformation. We investigated intramolecular polar contacts within the prodomain using PyMOL. For 10 of 23 variants different intramolecular contacts compared to the wildtype were predicted (Table 2).

**Table 2:** Overview of the different investigated prodomain variants and their polar contacts compared to wildtype DSC2.

Variant	Polar contacts wildtype	Polar contacts variant	Altered bonding	Plasma-Membrane localization
p.D30N	p.K107*	/	−	✓
p.C32S	p.D30		+	XX
p.C32A	p.D30		+	XX
p.V52A	p.L54, p.G77		X	✓
p.V52I	p.L54, p.G77		X	✓
p.V52G	p.L54, p.G77		X	XX
p.V52l	p.L54, p.G77	p.G77	−	✓
p.C57S	p.L54	p.L54 (2×), p.N53, p.K107	+++	✓
p.C57A	p.L54	p.L54, p.K107	+	✓
p.F71Y	p.S66, p.D68	p.D68	−	✓
p.F71A	p.S66, p.D68	p.D68	−	XX
p.F71S	p.S66, p.D68	p.D68	−	XX
p.G77V	p.L74, p.V52		X	XX
p.G77D	p.L74, p.V52		X	XX
p.G77S	p.L74, p.V52		X	XX
p.V79A	p.V49 (2×), p.G50		X	✓
p.V79I	p.V49 (2×), p.G50		X	✓
p.V79G	p.V49 (2×), p.G50	p.V49 (1×), p.G50	−	XX
p.V79l	p.V49 (2×), p.G50		X	✓
p.I96V	p.I109 (2×)		X	✓
p.I96T	p.I109 (2×)		X	✓
p.I96l	p.I109 (2×)		X	✓
p.I96A	p.I109 (2×)		X	✓

*An interaction to p.K107 could be found in the AlphaFold database but not modelled in PyMOL, **+**, one additional polar contact; - one deleted polar contact; **X,** no change; bold variants are ACM-related variants; **XX**, disturbed plasma membrane localization.

**Figure 7:**
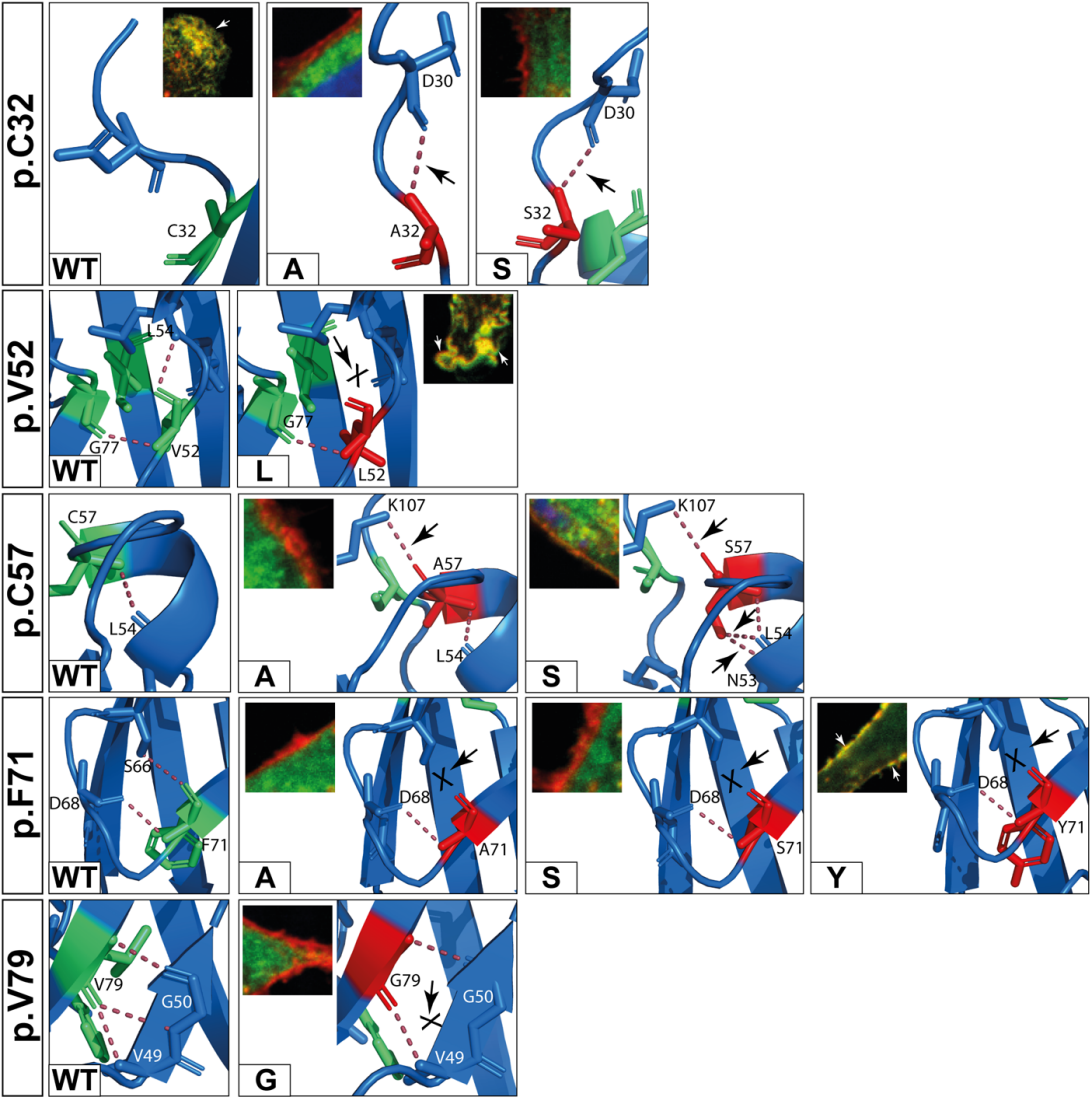
*In silico* predictions of polar contacts (red dashed line, arrow) in wildtype DSC2 compared to different prodomain variants. Variants that are not shown do not show different polar contacts *in silico* (see also Table 2). Conserved amino acids are shown in green and variants in red. Interacting amino acids are shown as sticks and are labeled. Intramolecular bonds are marked with a black arrow. The loss of a polar contact is marked with “X”. Fluorescence microscopic representative images show DSC2 prodomain variants (green) and Wheat germ agglutinin (WGA) conjugated with Alexa Fluor 633 as plasma membrane marker (red) in HT-1080 cells. Colocalization of DSC2 prodomain variants and WGA are indicated by white arrows. The variants p.V52l, p.C57A/S and p.F71Y show different intradomain contact sites but were correctly integrated in the plasma membrane. Five variants (p.C32A/S, p.F71A/S, p.V79G) were not integrated in the plasma membrane. One of the variants (p.V79G) is listed in the Human Gene Mutation Database (HGMD) and was not localized at the plasma membrane. In summary, changes of the molecular modeling structures do not correlate with the plasma membrane transport in the investiaged DSC2 constructs.

Five variants (p.C32S/A, p.F71A/S, p.V79G) showed aberrant intramolecular polar contacts and significantly reduced plasma membrane localization (Figures 4A, 7 and Table 2).

In contrast, five variants (p.D30N, p.V52l, p.C57A/S, p.F71Y) showed different intramolecular bonds but were found to the plasma membrane (Figures 4A,C, 7 and Table 2).

Four variants (p.V52G, p.G77V/D/S) did not reveal aberrant polar contacts but were nevertheless not found at the plasma membrane.

Finally, the two DSC2 variants p.V79A and p.I96T did not reveal aberrant intramolecular polar contacts and are poorly transported to the plasma membrane (Figure 4C).

### 3.8. Cadherins are transported to plasma membrane independent from the used cell line

To investigate whether the effects of the different prodomain variants are dependent from the cell type we transiently transfected HT-1080 cells and hiPSC-derived cardiomyocytes. The immunofluorescence microscopy revealed that the cadherin is found at the plasma membrane to a higher degree in hiPSC-derived cardiomyocytes but the data obtained in HT-1080 cells for different variants were comparable (Figure 4C). Therefore, we suggest that the processing of DSC2 is a general biological mechanism which is cell type independent.

## 4. Discussion

Variants in genes encoding proteins of the cardiac desmosomes are associated with ACM (57, 58). More than 1,600 variants are listed in genes encoding the cardiac desmosomal cadherins DSG2 and DSC2 in public genetic databases. However, for more than 75% of the listed variants within the extracellular domains of DSG2 and DSC2 the significance is unknown (29–32).

At present >75 entries (incl. 56 missense variants) are found within the DSC2 prodomain in the genetic disease databases HGMD (30), ClinVar (31), the ARVC database (29) as well as the human genetic population database gnomAD (32). 17% of the ClinVar-listed DSC2 variants were classified as (likely) pathogenic (27) and 11% were registered with conflicting data. The pathomechanisms of most variants remain unclear but may be relevant for disease classification (27) and for the understanding of the cellular cadherin processing.

In the context of ACM, adherence defects of the cardiac desmosome are relevant for disease development (59, 60). Therefore, the interactions of mutant extracellular cadherin domains were investigated before (13, 25, 26, 39). The cadherin *trans*-interaction is based on a strand-swap mechanism between the EC1 domains (13, 61–63). However, not only altered binding properties of the cadherins but also problems of the post-translational processing of the cadherins may be of pathogenic relevance (18, 20, 25, 38). The variants p.R46Q and p.R49H in DSG2 have an impact on the cleavage of the propeptide (38, 39, 64, 65). A proper plasma membrane localization of DSG2-p.R46Q was observed (38) but the adhesive properties were affected (39).

In contrast to DSG2, DSC2 and other classical cadherins carry an N-terminal prodomain lacking adhesive properties (Supplementary Figure S3) (14). The prodomains of cell-cell adhesion proteins in general may have protective functions during the maturation process by preventing folding into the biological active form and/or blocking premature adhesion (3, 4, 14). It was reported recently that the precursor of the classical N-cadherin reaches the cell surface but remains non-adhesive (66). Little is known on the disease mechanisms of DSC2 variants within its prodomain. Beffagna et al. found that the variant DSC2-p.E102K was not transported to the PM (67). Furthermore, the two variants p.R203C in the EC1 and p.T275M (EC2) were not properly processed (68). Thus, proper processing of the DSC2 N-terminus may be of pathogenic impact.

Here, we investigated the effects of nine ACM related missense variants of the DSC2 prodomain listed in disease databases and 14 variants of phylogenetically conserved amino acids. We analyzed the transport to the plasma membrane of DSC2 constructs carrying different signal peptides or lacking the prodomain. DSC2 with the wildtype signal peptide but lacking the prodomain remained in the ER and Golgi. In contrast, we found that the DSC2 constructs without the prodomain were transported to the plasma membrane if signal peptides of IgG or Sec were used.

Of note, all DSC2 prodomain constructs carrying the ACM variants p.G77V/S/D and p.V79G were not found at the plasma membrane, whereas p.D30N, p.V52A/I and p.I96V localization was comparable to the wildtype. The membrane distribution of the ACM-associated variant p.I96T and the model variants p.C57A/S and p.V79A was not different from the wildtype but seemed to be poorly transported. Moreover, we found evidence in the secretion assay that the prodomain of the variant p.V79G was not cleaved. We conclude from those experiments that the DSC2 prodomain is crucial for the transport mechanism of DSC2 and some of the ACM related variants interfere with the cadherin transport. Additional experimental model variants indicate that not only the position of the variant but the type of amino acid exchange appears to be relevant for the intracellular transport mechanism of DSC2.

The variant p.V79G was previously classified as pathogenic in a 73-year-old male ACM patient (34). He presented with sustained ventricular tachycardia aged 47. The pedigree revealed two first degree relatives who died from sudden cardiac death with unknown genotype. Therefore, the variant is considered today as a VUS according to the ACMG-guidelines. Our *in vitro* data indicate a significant effect on the intracellular cadherin transport, which may cause functional insufficiency.

Of note, we and others found previously that homozygous truncating variants lacking the transmembrane domains of DSG2 or DSC2 are associated with a severe ACM-phenotype (18–20), whereas heterozygous carriers remained unaffected. This suggests that truncating variants in DSG2 and DSC2 lead to recessive forms of ACM (18–20). Therefore, we suggest that the disease related DSC2 variants interfering with the plasma membrane localization may show a clinical phenotype preferentially when both alleles are affected. This is in line with the recent classification of the heterozygous variant DSC2-p.E102K (rs144799937) (67), which was also not found at the plasma membrane, as likely benign.

The amino acids DSC2-p.D30 and -p.C32 are located near the predicted signal peptidase cleavage site (40). DSC2-p.D30N is listed in disease databases (rs1987673824) but did not influence the localization *in vitro*. However, exchange of p.C32 which is part of the predicted cleavage site against alanine (A) or serine (S) blocked the plasma membrane localization of DSC2. Based on our experimental data we cannot determine an *in vitro* effect of the variant p.D30N but identified that p.C32 is a critical N-terminal residue for the intracellular transport of DSC2.

An *in silico* structure prediction model of the prodomain revealed an extracellular cadherin like domain with prominent β-sheets in DSC2 comparably to the N-cadherin prodomain (14). The conserved amino acids appear to stabilize the prodomain mainly by polar bonds. Although some variants led to loss of intradomain bonding (p.D30N, p.V52l, p.F71Y/A/S, p.V79G), in others additional contacts were predicted (p.C32A/S, p.C57A/S) or the intradomain bonds remained unaffected (p.V52A/I/G, p.G77V/D/S, p.V79A/I/L, p.I96V/T/L/A). We did not find any *in silico* evidence for major structural differences between the wildtype and the mutant prodomains explaining the observed differences of the intracellular cadherin transport (compare Supplementary Figure. S4).

It was previously published that plakoglobin (JUP) as a direct binding partner of DSC2 can be relocalized in patients diagnosed with an ACM. Although immunohistochemical evidence of the JUP relocalization within the intercalated disc is conflicting (20, 69, 70) we performed staining of JUP and connexin-43 (CX43) in hiPSC-derived cardiomyocytes. Transfection of wildtype DSC2 and the variant p.V79G in hiPSC-cardiomyocytes revealed no differences when stained for JUP and CX43, *in vitro* (Supplementary Figure S5). This confirmed previous data from *in vivo* stainings in human ARVC (69).

In conclusion, we found that signal peptide and prodomain of DSC2 are critical for the transport of the cadherin to the plasma membrane. Intracellular DSC2 transport is possible with constructs containing different signal peptides and even without the prodomain. Some constructs with variants at conserved amino acid positions within the prodomain are also not transported to the cell surface. Thus, the prodomain and specific amino acids are indispensable for proper maturation and intracellular transport of DSC2. Some but not all ACM related amino acid exchanges within the prodomain have impact on the transport and cleavage of the prodomain. Here, we provide *in vitro* data on the molecular pathomchanisms of DSC2 variants which may support evidence classification in clinical genetics.

## 5. Limitations

We presented *in vitro* data evaluated in different cell culture systems. We mapped critical amino acid residues within the prodomain of DSC2 by transfection. We focused our read-out on the DSC2-localization at plasma membrane. We cannot exclude that other disease mechanisms like e.g., altered nanomechanical properties may play a role in those ACM related variants transported like the wildtype at the plasma membrane.

## Data Availability

The original contributions presented in the study are included in the article/**Supplementary Material**, further inquiries can be directed to the corresponding author.
