# Supplementary material for: Cardiomyopathy related desmocollin-2 prodomain variants affect the intracellular cadherin transport and processing
Source: Front Cardiovasc Med. 2023 May 19;10:1127261. doi: 10.3389/fcvm.2023.1127261 (PMC10235514; doi:10.3389/fcvm.2023.1127261)
Supplement: Supplementary file 1 [file Datasheet1.pdf]

Supplementary Material

Cardiomyopathy related desmocollin-2 prodomain variants affect the intracellular cadherin transport and processing

Greta Marie Pohl[1], Manuel Göz[2], Anna Gärtner[1], Andreas Brodehl[1], Tolga Cimen[3], Ardan M. Saguner[3], Eric Schulze-Bahr[4], Volker Walhorn[2], Dario Anselmetti[2], Hendrik Milting[1*]

*** Correspondence:**
Prof. Dr. Hendrik Milting (PhD), Erich & Hanna Klessmann-Institute for Cardiovascular Research and Development, Heart- and Diabetes Center NRW, University Hospital of the Ruhr-University Bochum, Georgstr. 11, D-32545 Bad Oeynhausen, Germany.
hmilting@hdz-nrw.de, Phone: +49-(0)5731-97 3510

1 Supplementary Figures

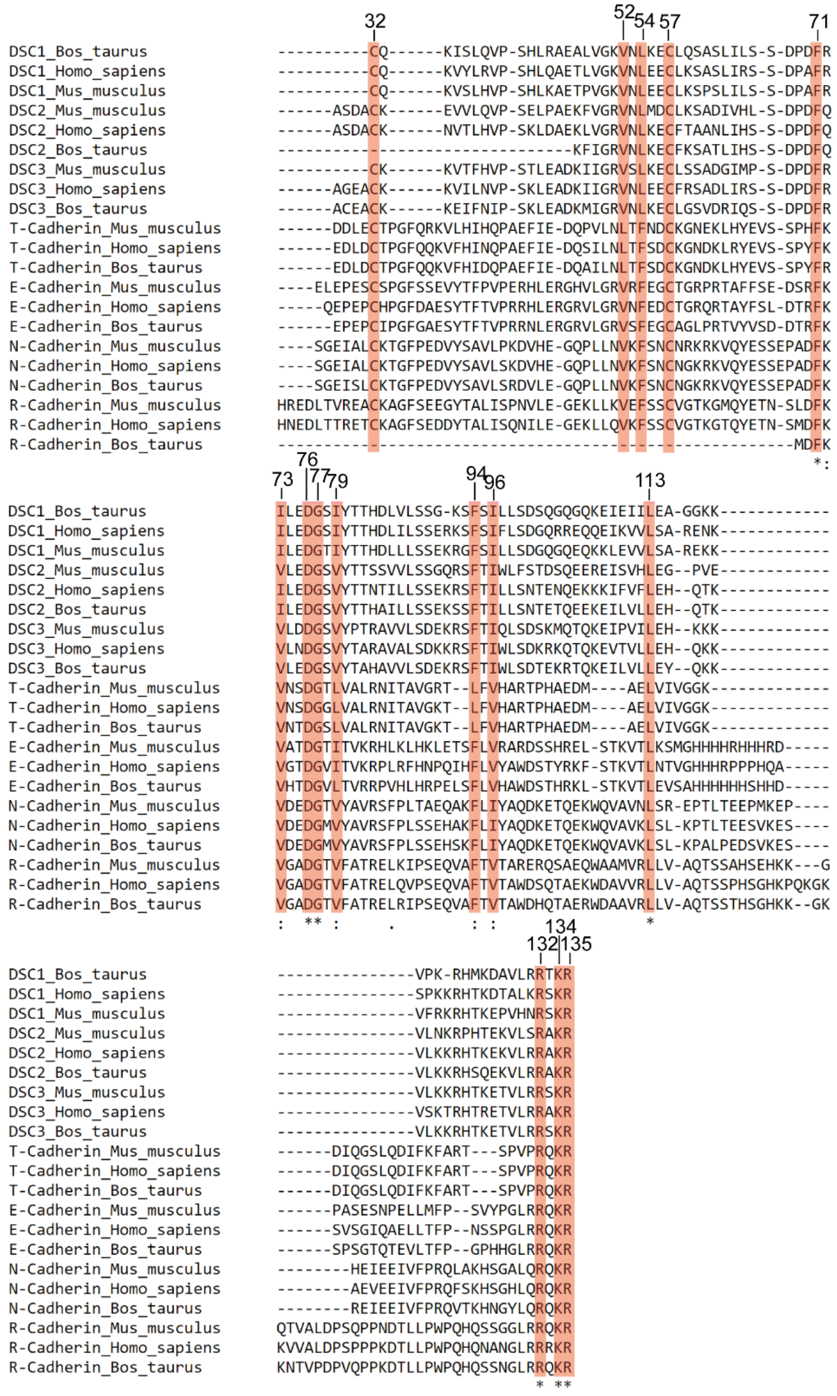

Supplementary Figure 1. Multiple amino acid alignment of the prodomains of DSC1-3 and different classic cadherins N-, R-, E-Cadherin and cadherin-like protein T-Cadherin from different species (*Homo sapiens*, *Bos taurus*, *Mus musculus*) using Clustal Omega (1). Sequences were obtained from UniProt Database (2). Conserved amino acids are marked with orange boxes.

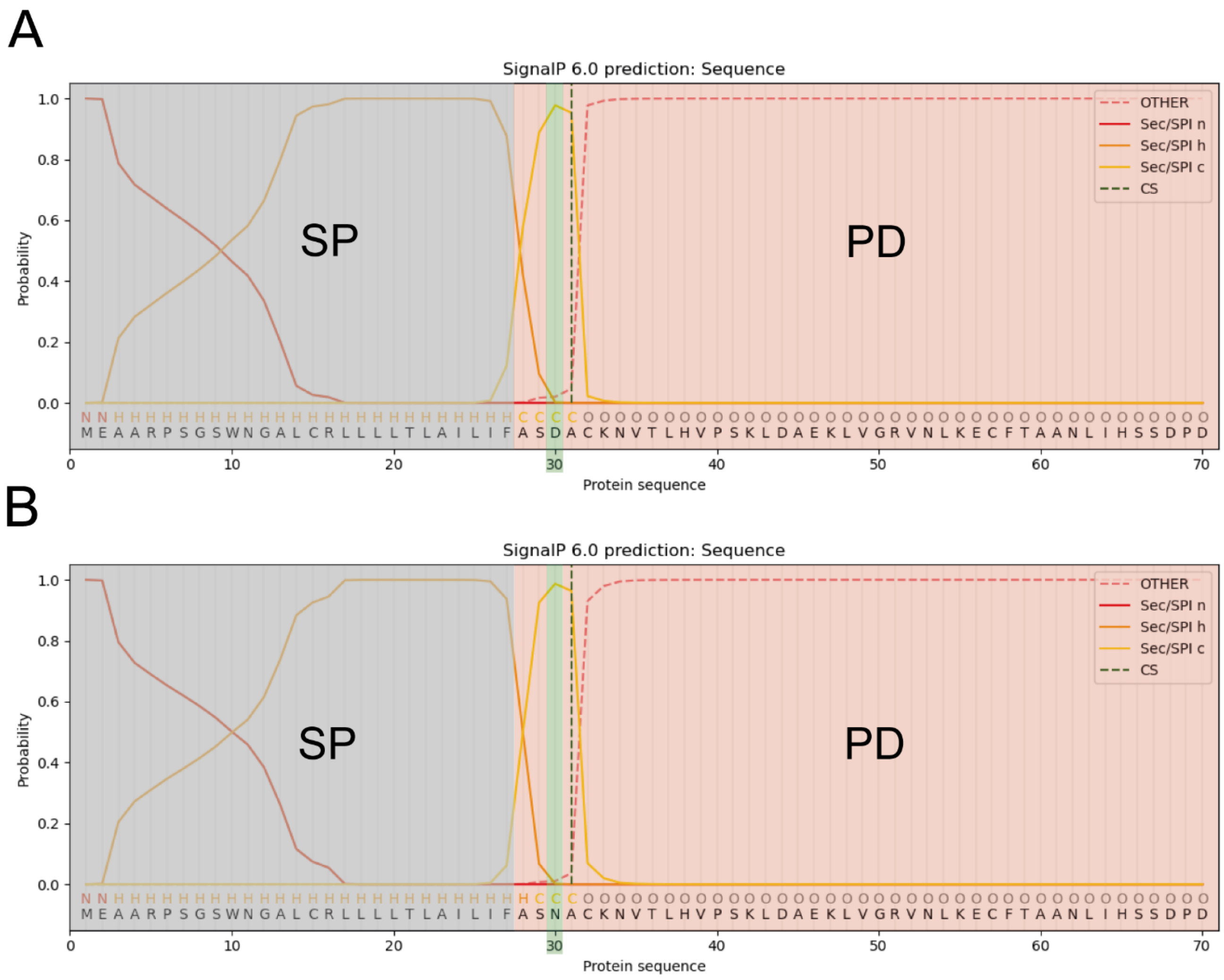

Supplementary Figure 2. Putative signal peptidase cleavage site for **(A)** wildtype DSC2 and **(B)** DSC2-p.D30N. The *in silico* prediction was done using SignalP 6.0 (3). Signal sequence cleavage site is predicted with 95% probability between positions p.A31 and p.C32. SP = Signal peptide (grey), PD = Prodomain (red). The position p.D30 is shown in green. The cleavage site is not affected by the substitution of p.D30N *in silico*.

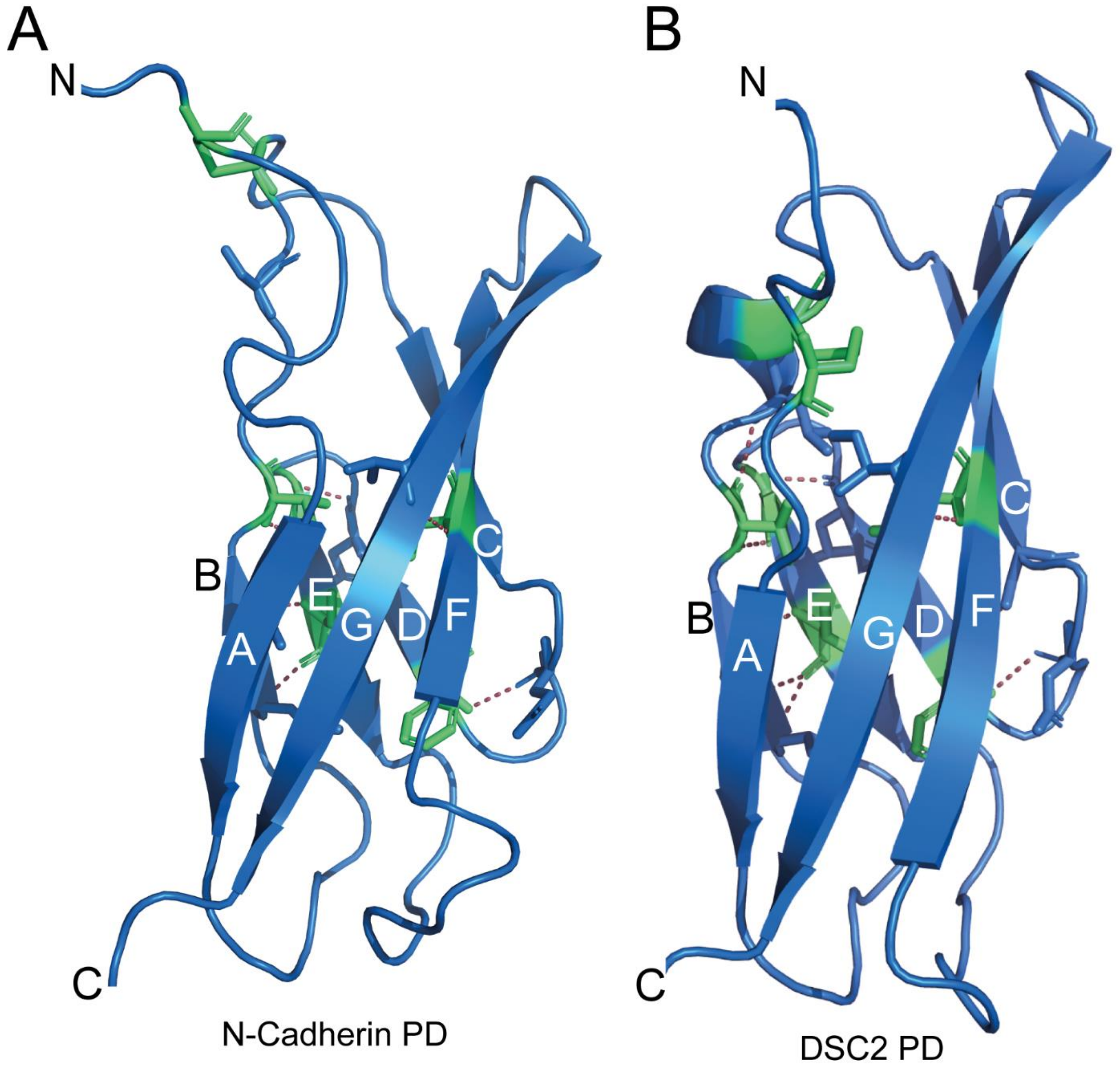

Supplementary Figure 3. (A) Ribbon presentation of the prodomain (PD) of the classical N-Cadherin (aa26-aa159) compared to the **(B)** *in silico* prediction of the wildtype DSC2 PD (aa28-aa135) (4, 5). Interacting and conserved amino acids (green) are shown as sticks. Polar contacts between amino acids from conserved positions are shown as red dashed lines. Designation of the β-sheets corresponds to those of Koch *et al*., 2004 (6). N- and C-termini are indicated.

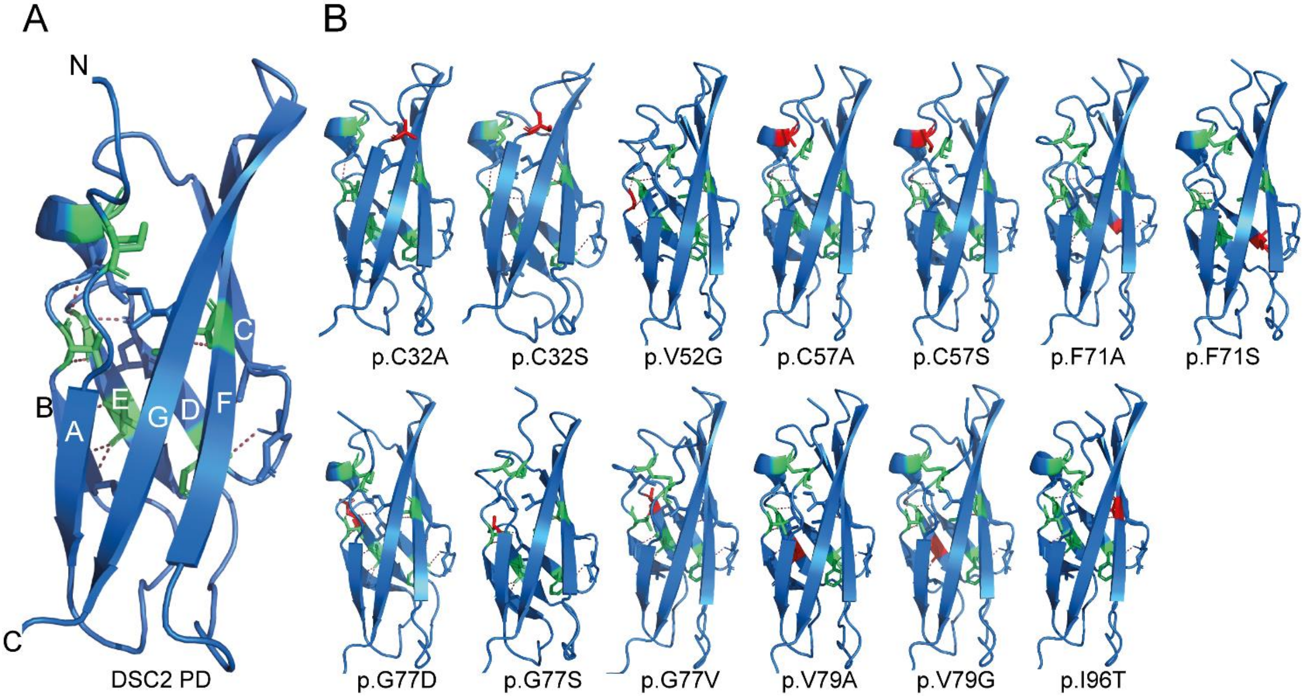

Supplementary Figure 4. Ribbon presentation of the *in silico* predictions of **(A)** the wildtype DSC2 prodomain (PD) (aa28-aa135) (4, 5). Designation of the β-sheets respond to those of Koch *et al*., 2004 (6). N- and C-termini are indicated. **(B)** Predicted ribbon structures of different variants (red) at conserved positions (green) within the DSC2 PD (7) that affect subcellular plasma membrane localization. Polar contacts are shown as red dashed lines. Interacting amino acids from conserved positions are shown as sticks.

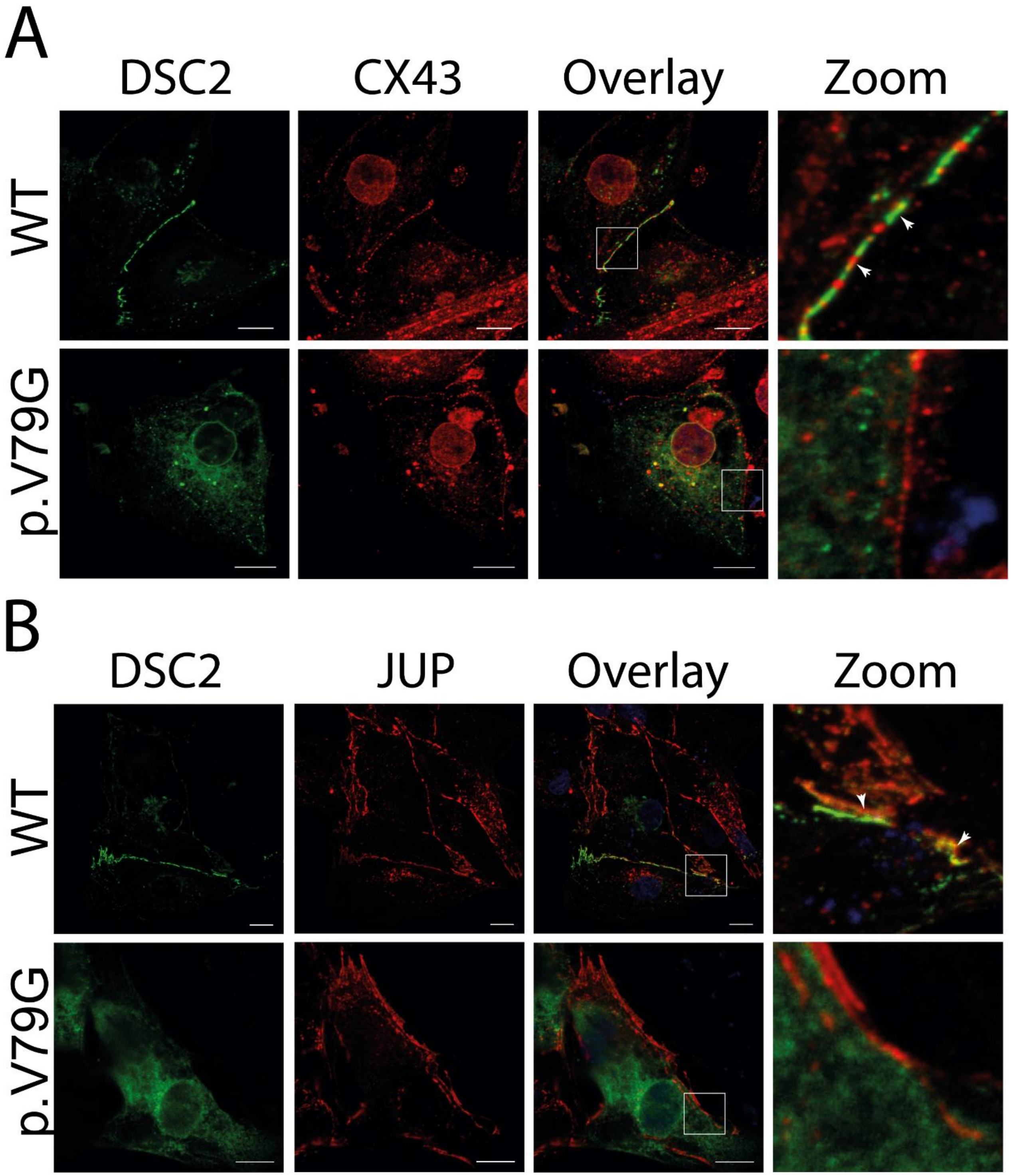

Supplementary Figure 5. Localization of **(A)** connexin-43 (CX43, #11370, 1:100 in 1% BSA/PBS, Abcam, red) and **(B)** plakoglobin (JUP, BD610253, 1:100 in 1% BSA/PBS, BD Biosciences, red) for DSC2 wildtype (WT, green) and a representative variant which is not properly localized at the plasma membrane (p.V79G) in human iPSC-derived cardiomyocytes (no ACTN2 staining). No *in vitro* influence on the localization of both, JUP and CX43, was observed for the variant p.V79G.

2 Supplementary Tables

Supplementary Table 1: Oligonucleotides that were used for site-directed mutagenesis in *DSC2*.

**Name**	**Sequence [5’-…-3’]**
D30N_fwd	GCGATCTTAATATTTGCCAGTAATGCCTGCAAAAATGTGACAT
D30N_rev	ATGTCACATTTTTGCAGGCATTACTGGCAAATATTAAGATCGC
C32A_fwd	ATCTTAATATTTGCCAGTGATGCCGCCAAAAATGTGACATTACATGTTCC
C32A_rev	GGAACATGTAATGTCACATTTTTGGCGGCATCACTGGCAAATATTAAGAT
C32S_fwd	TTAATATTTGCCAGTGATGCCTCCAAAAATGTGACATTACATGTTC
C32S_rev	GAACATGTAATGTCACATTTTTGGAGGCATCACTGGCAAATATTAA
V52A_fwd	CGAGAAACTTGTTGGTAGAGCTAACCTGAAAGAGTGCTTTA
V52A_rev	TAAAGCACTCTTTCAGGTTAGCTCTACCAACAAGTTTCTCG
V52I_fwd	CCGAGAAACTTGTTGGTAGAATTAACCTGAAAGAGTGCTTT
V52I_rev	AAAGCACTCTTTCAGGTTAATTCTACCAACAAGTTTCTCGG
V52G_fwd	CGAGAAACTTGTTGGTAGAGGTAACCTGAAAGAGTGCTTTA
V52G_rev	TAAAGCACTCTTTCAGGTTACCTCTACCAACAAGTTTCTCG
V52L_fwd	CGAGAAACTTGTTGGTAGACTTAACCTGAAAGAGTGCTT
V52L_rev	AAGCACTCTTTCAGGTTAAGTCTACCAACAAGTTTCTCG
C57A_fwd	TTGGTAGAGTTAACCTGAAAGAGGCCTTTACAGCTGCAAATCTAATTC
C57A_rev	GAATTAGATTTGCAGCTGTAAAGGCCTCTTTCAGGTTAACTCTACCAA
C57S_fwd	GTAGAGTTAACCTGAAAGAGTCCTTTACAGCTGCAAATCTAAT
C57S_rev	ATTAGATTTGCAGCTGTAAAGGACTCTTTCAGGTTAACTCTAC

F71Y_fwd	CAAATCTAATTCATTCAAGTGATCCTGACTACCAAATTTTGGAGGAT
F71Y_rev	ATCCTCCAAAATTTGGTAGTCAGGATCACTTGAATGAATTAGATTTG
F71A_fwd	TAATTCATTCAAGTGATCCTGACGCCCAAATTTTGGAGGATGGTTCAG
F71A_rev	CTGAACCATCCTCCAAAATTTGGGCGTCAGGATCACTTGAATGAATTA
F71S_fwd	CAAATCTAATTCATTCAAGTGATCCTGACTCCCAAATTTTGGAGGAT
F71S_rev	ATCCTCCAAAATTTGGGAGTCAGGATCACTTGAATGAATTAGATTTG
G77V_fwd	TGACTTCCAAATTTTGGAGGATGTTTCAGTCTATACAACAAATACTA
G77V_rev	TAGTATTTGTTGTATAGACTGAAACATCCTCCAAAATTTGGAAGTCA
G77D_fwd	TGACTTCCAAATTTTGGAGGATGATTCAGTCTATACAACAAATACTA
G77D_rev	TAGTATTTGTTGTATAGACTGAATCATCCTCCAAAATTTGGAAGTCA
G77S_fwd	CTGACTTCCAAATTTTGGAGGATAGTTCAGTCTATACAACAAATACT
G77S_rev	AGTATTTGTTGTATAGACTGAACTATCCTCCAAAATTTGGAAGTCAG
V79G_fwd	ATAGAATAGTATTTGTTGTATAGCCTGAACCATCCTCCAAAATTTGG
V79G_rev	CCAAATTTTGGAGGATGGTTCAGGCTATACAACAAATACTATTCTAT
V79A_fwd	CCAAATTTTGGAGGATGGTTCAGCCTATACAACAAATACTATTCTAT
V79A_rev	ATAGAATAGTATTTGTTGTATAGGCTGAACCATCCTCCAAAATTTGG
V79I_fwd	ACTTCCAAATTTTGGAGGATGGTTCAATCTATACAACAAATACTATTC
V79I_rev	GAATAGTATTTGTTGTATAGATTGAACCATCCTCCAAAATTTGGAAGT
V79L_fwd	CTTCCAAATTTTGGAGGATGGTTCACTCTATACAACAAATACTATT
V79L_rev	AATAGTATTTGTTGTATAGAGTGAACCATCCTCCAAAATTTGGAAG

I96V_fwd	CGGAGAAGAGAAGTTTTACCGTATTACTTTCCAACACTGAG
I96V_rev	CTCAGTGTTGGAAAGTAATACGGTAAAACTTCTCTTCTCCG
I96T_fwd	CGGAGAAGAGAAGTTTTACCACATTACTTTCCAACACTGAGAA
I96T_rev	TTCTCAGTGTTGGAAAGTAATGTGGTAAAACTTCTCTTCTCCG
I96L_fwd	CGGAGAAGAGAAGTTTTACCCTATTACTTTCCAACACTGAG
I96L_rev	CTCAGTGTTGGAAAGTAATAGGGTAAAACTTCTCTTCTCCG
I96A_fwd	CTCGGAGAAGAGAAGTTTTACCGCATTACTTTCCAACACTGAGAAC
I96A_rev	GTTCTCAGTGTTGGAAAGTAATGCGGTAAAACTTCTCTTCTCCGAG
delSP_fwd (pCEP4)	CGCCGCCACCATGGCCAGTGATGCCT
delSP_rev (pCEP4)	AGGCATCACTGGCCATGGTGGCGGCG
delSP_fwd (peYFP)	CTCAGATCTCGAGATGGCCAGTGATGCCTGCA
delSP_rev (peYFP)	TGCAGGCATCACTGGCCATCTCGAGATCTGAG
delPD_fwd	GCTGACCCTCGCGATCTTAATATTTAGATGGGCTCCAA
delPD_rev	TTGGAGCCCATCTAAATATTAAGATCGCGAGGGTCAGC
Sec-PD_fwd	ACTCAGATCTCGAGATGTGGTGGCGCCTGTGGTGGCTGCTGCTGCTGC TGCTGCTGCTGTGGCCCATGGTGTGGGCCGCCAGTGATGCCTGC
Sec-PD_rev	GTGGCGACCGGTCCTCTC
Sec-delPD_fwd	ACTCAGATCTCGAGATGTGGTGGCGCCTGTGGTGGCTGCTGCTGCTGCTG CTGCTGCTGTGGCCCATGGTGTGGGCCAGATGGGCTCCAATTCCTTGTTC

Sec-delPD_rev	GTGGCGACCGGTCCTCTC
Sec-D30N_fwd	GTGTGGGCCGCCAGTAATGCCTGCAAAAATG
Sec-D30N_rev	CATTTTTGCAGGCATTACTGGCGGCCCACAC
IgG-PD_fwd	ACTCAGATCTCGAGATGGAGTTTGGACTGAGCTGGCTTTTTCTTGTGGCTT TTTTAAAAGGTGTCCAGTGTGCCAGTGATGCCTGC
IgG-PD_rev	GTGGCGACCGGTCCTCTC
IgG-delPD_fwd	ACTCAGATCTCGAGATGGAGTTTGGACTGAGCTGGCTTTTTCTTGTGGCTT TTTTAAAAGGTGTCCAGTGTAGATGGGCTCCAATTCCTTGTTC
IgG-delPD_rev	GTGGCGACCGGTCCTCTC
IgG-D30N_fwd	GTGTCCAGTGTGCCAGTAATGCCTGCAAAAATGTG
IgG-D30N_rev	CACATTTTTGCAGGCATTACTGGCACACTGGACAC

1. Madeira F, Pearce M, Tivey ARN, Basutkar P, Lee J, Edbali O, et al. Search and sequence analysis tools services from EMBL-EBI in 2022. Nucleic Acids Res. 2022;50(W1):W276-W9.

2. UniProt C. UniProt: the universal protein knowledgebase in 2021. Nucleic Acids Res. 2021;49(D1):D480-D9.

3. Teufel F, Almagro Armenteros JJ, Johansen AR, Gislason MH, Pihl SI, Tsirigos KD, et al. SignalP 6.0 predicts all five types of signal peptides using protein language models. Nat Biotechnol. 2022;40(7):1023-5.

4. Jumper J, Evans R, Pritzel A, Green T, Figurnov M, Ronneberger O, et al. Highly accurate protein structure prediction with AlphaFold. Nature. 2021;596(7873):583-9.

5. Varadi M, Anyango S, Deshpande M, Nair S, Natassia C, Yordanova G, et al. AlphaFold Protein Structure Database: massively expanding the structural coverage of protein-sequence space with high-accuracy models. Nucleic Acids Res. 2022;50(D1):D439-D44.

6. Koch AW, Farooq A, Shan W, Zeng L, Colman DR, Zhou MM. Structure of the neural (N-) cadherin prodomain reveals a cadherin extracellular domain-like fold without adhesive characteristics. Structure. 2004;12(5):793-805.

7. Mirdita M, Schutze K, Moriwaki Y, Heo L, Ovchinnikov S, Steinegger M. ColabFold: making protein folding accessible to all. Nat Methods. 2022.
